# The Skin Microbiome and Bioactive Compounds: Mechanisms of Modulation, Dysbiosis, and Dermatological Implications

**DOI:** 10.3390/molecules30224363

**Published:** 2025-11-11

**Authors:** Katarzyna Wojciechowska, Katarzyna Dos Santos Szewczyk

**Affiliations:** 1 Chair and Department of Applied Pharmacy, Medical University of Lublin, Chodźki 1, 20-093 Lublin, Poland; 2 Department of Pharmaceutical Botany, Medical University of Lublin, Chodźki 1, 20-093 Lublin, Poland; katarzyna.dossantos-szewczyk@umlub.edu.pl

**Keywords:** skin microbiome, dysbiosis, bioactive compounds, natural products, prebiotics, probiotics, postbiotics, gut–skin axis, dermatology, cosmetology

## Abstract

Maintaining the balance between the host and commensal microorganisms is essential for skin health. The disruption of this equilibrium (dysbiosis) can contribute to inflammatory and infectious diseases and accelerate skin aging. Dysbiosis also accompanies skin cancers and may influence their progression. Causes of dysbiosis include exogenous factors such as cosmetics, UV radiation, pollution, and diet, as well as endogenous factors like stress, hormonal imbalances, and aging. Standard antibacterial treatments often eliminate beneficial microbes and may exacerbate conditions. Consequently, there is growing interest in alternative strategies—notably natural plant- and animal-derived products—that can modulate the skin microbiome more selectively and gently. This review presents current knowledge on skin microbiome physiology and dysbiosis and discusses natural compounds and microbiome-based therapies (probiotics, prebiotics, postbiotics) that modulate the skin microbiota. Unlike prior reviews, we provide a comparative perspective on emerging compound classes (e.g., peptides, lipids) and integrate the skin–gut axis concept into the framework, highlighting mechanistic insights at molecular and clinical levels. Our synthesis emphasizes distinct modes of action and evidence levels—from in vitro mechanisms to clinical outcomes—and offers guidance for formulation of microbiome-compatible products.

## 1. Introduction

The human microecosystem consists of microbial communities colonizing the oral The human microecosystem comprises microbial communities on the skin, oral cavity, gut, and other sites [1]. The skin microbiome differs by body location, reflecting humidity, sebum production, and other factors [2,3]. It includes bacteria (mostly *Actinobacteria*, *Firmicutes*, *Proteobacteria*, *Bacteroidetes*), fungi (e.g., *Malassezia*), viruses, and mites (*Demodex* spp.) [2,3,4,5,6,7]. Dominant species such as *Staphylococcus epidermidis*, *Cutibacterium acnes*, and *Corynebacterium* interact closely with the host [2,3,5,7]. Commensals induce keratinocytes to produce antimicrobial peptides (AMPs) that selectively target pathogens [5,6], activate the aryl hydrocarbon receptor (AhR) to support barrier repair [8], and contribute to lipid matrix formation. For example, *S. epidermidis* produces sphingomyelinase which generates ceramides and reduces transepidermal water loss (TEWL) [5,9]. Nonetheless, commensals can become opportunistic pathogens under certain conditions [2]. Microbiome composition is also shaped by age, sex, ethnicity, hormones, lifestyle, and hygiene [1,7]. Disruption of the skin–microbe balance (dysbiosis) is implicated in acne, atopic dermatitis (AD), chronic wounds, psoriasis, and other disorders [6,8,10]. Conventional therapies (antibiotics, antiseptics) reduce pathogens but also eliminate beneficial taxa and promote resistance [7]. This has stimulated interest in natural products (botanicals, bee products, chitosan, etc.) and microbiome-based therapies that selectively modulate the skin microbiota while supporting homeostasis [7,8,11].

A key novelty of this review is its comprehensive integration of multiple emerging topics. We explicitly compare modes of action across bioactive classes—including peptides and lipids, which have received less attention—and incorporate the gut–skin axis. The gut–skin axis recognizes that gut microbes and their metabolites (e.g., SCFAs) can enter the circulation to influence systemic immunity and directly affect the skin barrier and inflammation [12]. For instance, gut-derived short-chain fatty acids (SCFAs) regulate host immune tone and keratinocyte function, and gut dysbiosis can contribute to systemic inflammation that alters skin homeostasis [12]. We also organize evidence by experimental context (in vitro, animal models, clinical studies) and highlight formulation factors (dose, vehicle, pH).

To compile this review, a comprehensive literature search was conducted following PRISMA guidelines. Databases including PubMed, Web of Science, Scopus, and Google Scholar were searched (through August 2025) using terms such as “skin microbiome”, “dysbiosis”, “prebiotic”, “probiotic”, “postbiotic”, “bioactive”, “dermatology”, “cosmetics”, along with specific compound and condition keywords (e.g., “polyphenol”, “SCFA”, “atopic dermatitis”). We included English-language original research and review articles focused on skin microbiome modulation by bioactive compounds. References of retrieved papers were screened to identify additional studies. Articles solely on non-skin microbiomes (gut, oral, etc.) or outside the scope were excluded. This narrative approach, guided by systematic methods, aims to capture current mechanistic and clinical insights in skin microbiome modulation.

## 2. Functions of the Skin Microbiome

The skin microbiome is essential for barrier integrity and immunity [13]. By occupying ecological niches, commensals compete with pathogens for space and nutrients. Many produce antimicrobial substances (bacteriocins, proteases, hydrogen peroxide) [13,14], that confer colonization resistance, especially against *Staphylococcus aureus*. For example, *S. epidermidis* not only inhibits pathogens but also stimulates keratinocytes to produce AMPs [15]. Commensals modulate immune responses: they induce keratinocytes to release cytokines (e.g., IL-1α) that help calibrate T-cell responses [13,16], and they promote immune tolerance to non-pathogenic microbes, preventing chronic inflammation [17,18].

Another key function is metabolism of skin lipids. Microbes degrade sebum components and secretions into free fatty acids and lactic acid, which maintain the acidic pH of the stratum corneum. Acidic pH restrains pathogens and favors commensals [13]. The microbiome also contributes to epidermal desquamation and the production of moisturizing factors, supporting the hydrolipid barrier [17,18]. Some skin bacteria (e.g., *S. epidermidis*) produce enzymes (like sphingomyelinase) that help the host generate ceramides for barrier function [19]. Indeed, certain skin lipids themselves have antimicrobial roles: for example, sapienic acid (a sebaceous free fatty acid) can effectively inhibit *S. aureus* [19]. Thus, the skin barrier and microbiome engage in reciprocal interactions to preserve homeostasis.

Clinical studies have shown that topical application of *S. epidermidis* to the forehead improves skin hydration and reduces TEWL [20], while the use of certain *S. hominis* strains can improve the skin condition of patients with atopic dermatitis (AD) and inhibit the growth of *S. aureus* [21,22]. Disturbances in the microbiome lead to dysbiosis, resulting in skin dryness, inflammation, and increased susceptibility to infections. Reduced sebaceous gland activity alters sebum composition, creating favorable conditions for pathogen colonization [23]. Several commensal bacterial species, particularly from the genera *Cutibacterium* and *Lactobacillus*, play a critical role in maintaining skin homeostasis by supporting the epidermal barrier and exhibiting anti-inflammatory and antioxidant properties. They produce metabolites such as AMPs and enzymes that participate in epidermal renewal. A decline in the abundance of commensal bacteria, accompanied by an overgrowth of potentially pro-inflammatory microorganisms such as *Corynebacterium* and *Proteobacteria*, observed in elderly individuals, contributes to barrier dysfunction, collagen degradation, and accelerated skin aging. Therefore, preserving the balance of the skin microbiome is considered one of the potential targets of modern anti-aging strategies [24].

## 3. Dysbiosis and Skin Disorders

Dysbiosis—an imbalance in microbial communities—is increasingly recognized in many skin diseases [16,17]. In acne, shifts favor pathogenic *C. acnes* strains and *Staphylococcus* spp. in inflamed follicles. Atopic dermatitis (AD) features reduced diversity, with overgrowth of *S. aureus* and loss of commensals like *S. epidermidis*. Psoriasis, rosacea, chronic wounds, and seborrheic dermatitis also show distinct dysbiotic patterns linked with barrier defects, biofilms, and immune skewing. Importantly, dysbiosis often correlates with clinical severity. For example, *S. aureus* abundance in atopic dermatitis correlates with itch and barrier dysfunction, whereas restoring microbial diversity can improve outcomes.

Traditional lists of dysbiosis-associated taxa are being replaced by mechanistic understanding: pathogen overgrowth (e.g., toxin-producing *S. aureus*), loss of biofilm-suppressing commensals, and altered metabolite profiles (e.g., reduced SCFAs) can all trigger skin inflammation. This review focuses on interventions that selectively modulate the microbiome rather than broadly killing bacteria.

### 3.1. Acne

The skin microbiota, particularly *C. acnes*, plays a crucial role in the pathogenesis of acne [13]. While traditionally considered a commensal resident of sebaceous glands in healthy skin, increasing evidence suggests that its contribution to acne development depends on the presence of specific strains and their genetic diversity. Strains belonging to ribotypes RT4 and RT5, especially of the IA1 phylotype, display strong virulent properties, including biofilm formation, production of proteolytic enzymes, lipases, porphyrins, and stimulation of Th17/Th1-type immune responses. These strains are thought to activate TLR2, TLR4, and CD36 receptors, inducing the release of pro-inflammatory cytokines such as IL-1β, TNF-α, IL-6, IL-8, and IL-17A, thereby driving inflammation and the formation of papules, pustules, and nodules [25,26]. Not all *C. acnes* strains are pathogenic. The RT6 strain is considered non-pathogenic and is associated with healthy skin. It contributes to maintaining the acidic pH of the epidermis by releasing free fatty acids and competes with pathogens for ecological niches, thereby promoting microbial homeostasis. A similar protective role is played by *S. epidermidis*, which produces AMPs (e.g., epidermin, lipoteichoic acids) that inhibit the growth of pathogens, including virulent *C. acnes* strains. In addition, in vitro studies have shown that *S. epidermidis* can suppress excessive inflammatory responses by modulating miRNA expression and TLR signaling in keratinocytes [26]. The acne-associated skin microbiota also includes yeasts of the genus *Malassezia*, which dominate in seborrheic areas. Although generally regarded as commensals, in some cases they contribute to the development of so-called “yeast acne” (*Malassezia folliculitis*), characterized by papules and pustules resistant to antibiotic therapy. Lipolytic enzymes of *Malassezia* spp. exhibit activity more than 100 times greater than the lipases of *C. acnes*, leading to excessive triglyceride breakdown in sebum and the generation of free fatty acids that irritate hair follicles. Some studies have demonstrated that antifungal therapy yielded superior clinical outcomes compared to antibiotic treatment [25]. Recent studies show that *C. acnes* contributes to acne not only through strain diversity and biofilm formation but also via extracellular vesicles (EVs) that amplify inflammation. EVs from phylotype IA1 strains isolated from inflammatory lesions trigger higher levels of IL-1β, IL-6, IL-8, IL-17α, IL-36γ, as well as hBD2 and LL-37, compared with EVs from healthy skin strains. This strain-dependent effect directly links microbial products to the intensified cytokine response in acne [27].

### 3.2. Atopic Dermatitis (AD)

AD is a prototypical dermatosis associated with skin microbiome dysbiosis. In healthy individuals, the skin surface is colonized by strains such as *S. epidermidis* and *S. hominis*, which produce potent AMPs. These natural antibiotics, including newly identified lantibiotics, selectively inhibit the growth of *S. aureus* and act synergistically with the human cathelicidin LL-37. In patients with AD, deficiencies of protective strains and their associated AMPs are frequently observed, which promotes increased colonization of the skin by *S. aureus* [22]. The skin microbiome in AD is characterized by markedly reduced biodiversity and often strong domination by *S. aureus* [28]. This pathogen produces superantigens, proteases, and toxins, including alpha-toxin, which damage the epidermal barrier, amplify the Th2 immune response, and stimulate IgE production, perpetuating the vicious cycle of allergic inflammation. In samples with a high abundance of *S. aureus*, a concomitant decline in protective bacteria such as *C. acnes* and *S. hominis* is observed [29]. Furthermore, a significantly reduced presence of *Malassezia* spp., typically abundant in healthy skin, has been reported on the neck of AD patients [29]. Importantly, studies have shown that transplantation of skin microbiota from healthy donors can alleviate AD symptoms. These findings suggest that dysbiosis in AD results not only from pathogenic overgrowth but also from the loss of protective commensal microorganisms [22]. Recent findings indicate that *S. aureus* contributes to AD not only through its abundance but also via strain-level diversity and microevolution. Variations in functional genes and the presence of mobile genetic elements, particularly prophages, enhance the adaptability, virulence, and antibiotic resistance of certain strains. These insights highlight the importance of strain-specific approaches and the potential of advanced genomic tools, such as whole-genome sequencing and machine learning, to support more precise diagnostics and personalized therapies in AD [30].

### 3.3. Psoriasis

The skin microbiome also plays a protective role in preventing and modulating inflammatory processes associated with psoriasis. Stehlikova et al. [31] observed that the skin microbiome of psoriatic patients contained higher proportions of *Streptococcus* spp. and *Malassezia* spp., along with relatively low levels of *Cutibacterium*, compared to healthy controls. Experimental data in animal models indicate that the skin microbiome plays a pivotal role in promoting the development and activity of regulatory T cells (Tregs), which maintain immunological tolerance to resident flora and protect against excessive inflammation [32].

Moreover, a literature analysis summarized by Celoria et al. [33] highlights that this mechanism may be a key element in the pathogenesis and therapy of inflammatory skin diseases, including psoriasis. Disturbances in this balance can lead to dendritic cell activation and increased Th17 responses. It has been demonstrated that probiotics such as *Lactobacillus* and *Bifidobacterium*, as well as postbiotics, can support immunomodulation and skin barrier regeneration, indicating the therapeutic potential of microbiome-based strategies in psoriasis [32,33]. The gut microbiome has also been implicated in the disease’s pathogenesis through the skin–gut axis, where microbial metabolites and bacteria can translocate into the bloodstream and influence cytokines such as IL-17 and IL-10 [32].

### 3.4. Rosacea

Rosacea is a chronic inflammatory dermatosis in which increasing importance has been attributed to skin microbiome disturbances. Dysbiosis can lead to excessive activation of the immune system and initiation of the chronic inflammatory response characteristic of this condition [34,35]. Patients frequently exhibit reduced levels of *C. acnes*, which may favor colonization by potentially pathogenic bacteria and by the mite *Demodex* [34].

One of the key factors associated with rosacea pathogenesis is excessive colonization of the skin by *Demodex folliculorum*. Under balanced biological conditions, these mites are considered commensals; however, overgrowth can cause mechanical damage to hair follicles and activation of TLR-2 receptors, initiating an inflammatory cascade mediated by pro-inflammatory cytokines [35]. *Demodex* may also act as a vector for bacteria such as *Heyndrickxia oleronia* (basonym *Bacillus oleronius*), which further stimulate immune responses by inducing cytokine secretion and neutrophil recruitment [34]. In conditions of dysbiosis, even *S. epidermidis* may exhibit pathogenic properties by producing increased amounts of proteins and virulence factors, contributing to the formation of papules and pustules [34]. Elevated skin temperature, observed in patients during active inflammation, further promotes the proliferation of *D. folliculorum* and may enhance the metabolism of pathogenic microorganisms. Environmental factors and damage to the epidermal barrier also contribute to microbiome disturbances. The skin of patients with rosacea additionally shows excessive expression of the cathelicidin LL-37 and elevated kallikrein-5 activity, which under physiological conditions serve defensive functions but in this disease lead to a sustained inflammatory state [35]. Although some reports suggest the presence of *C. acnes* in the skin of rosacea patients, more recent analyses indicate rather a reduced abundance or altered phylotype structure, which may compromise the protective role of this species [35].

### 3.5. Chronic Wounds

In chronic wounds, including diabetic foot ulcers (DFU), the microbiome plays a critical role in the healing process. According to Byrd et al. [13], more than half of DFU become infected, and the composition of the microbiota varies depending on wound depth and duration. In superficial wounds, *S. aureus* is usually the dominant pathogen, whereas chronic wounds show an increased presence of *Proteobacteria* [13]. A major challenge is the formation of biofilms, produced by both bacteria and fungi, e.g., *Candida albicans*, which hinder pathogen clearance, increase resistance to antibiotics and antiseptics, and perpetuate chronic inflammation.

Studies indicate that commensals can aid in pathogen clearance, maintain low skin pH, and limit biofilm development. For example, *Corynebacterium accolens* produces free fatty acids with antibacterial activity [13]. Chronic wounds and ulcers, particularly in elderly and diabetic patients, often contain biofilms formed by *S. aureus* and *P. aeruginosa*, accompanied by a lack of protective flora, which significantly impedes healing [13,17]. There is also evidence that *S. epidermidis* may facilitate wound healing by reducing inflammation [36].

Recent reviews emphasize that chronic wounds are sustained not only by microbial biofilms but also by persistent activation of the innate immune system, which maintains low-grade, non-resolving inflammation [37]. Dysregulated interactions between biofilm-forming bacteria and host immune cells, such as neutrophils and macrophages, are increasingly recognized as key drivers of delayed healing [37]. Furthermore, in vitro and in vivo studies confirm that biofilm-associated bacteria are often tolerant and resistant to antibiotics and antiseptics, which explains many clinical treatment failures [38]. Novel therapeutic strategies under development include enzymes degrading the biofilm matrix, quorum-sensing inhibitors, and immunomodulatory approaches, which may restore healing when conventional therapies are insufficient [38].

### 3.6. Seborrheic Dermatitis (SD) and Dandruff

Seborrheic dermatitis (SD) and dandruff are associated with the overgrowth of yeasts of the genus *Malassezia* [39]. Most commonly, an increased proportion of *M. restricta* is observed, with a concomitant decrease in *M. globosa*. This imbalance is linked to enhanced production of irritating and pro-inflammatory metabolites, such as unsaturated fatty acids and indoles (e.g., indole-3-carbaldehyde), which activate immune receptors (e.g., AhR), disrupt epidermal barrier function, and exacerbate inflammation [40,41]. At the same time, an increased abundance of bacteria, particularly *S. epidermidis* and *S. aureus*, further weakens skin integrity by raising pH and enhancing TEWL. The presence of *Staphylococcus* has been shown to correlate positively with the severity of clinical symptoms such as scaling, pruritus, and erythema [40].

Commensal bacteria of the genus *Cutibacterium*, which normally dominate healthy seborrheic skin, are significantly reduced in affected areas. Their presence is associated with improved skin hydration, sebum homeostasis, and the ability to produce bacteriocins that inhibit *Staphylococcus* growth. Moreover, the coexistence of *Cutibacterium* and *Malassezia* appears to mitigate skin damage compared with conditions where *M. restricta* is present alone, suggesting a potential protective role of *Cutibacterium* [40].

### 3.7. Vitiligo

Vitiligo is a chronic skin disorder characterized by focal loss of pigmentation due to melanocyte depletion. The underlying mechanisms have not been fully elucidated; however, increasing evidence suggests a role of the skin microbiome. Dysbiosis and reduced microbial diversity have been demonstrated within vitiliginous lesions [42]. Compared with healthy skin, where *Corynebacterium* plays an important role, vitiliginous skin shows an increased abundance of members of the *Flavobacteriales*, *Gammaproteobacteria*, *and Flavobacteria*. These findings suggest that the vitiligo-associated microbiota has a distinct composition, and alterations in microbial communities may influence the course and persistence of the disease [42].

### 3.8. Alopecia Areata (AA)

The development of AA is influenced by genetic predisposition, environmental factors, and, as increasingly emphasized in the literature, by the skin and gut microbiome [43,44,45]. Imbalances in the hair follicle microbiome are associated with disrupted homeostasis, modulation of immune responses, and intensified peribulbar inflammation [2]. Pinto et al. [46] observed that patients with AA exhibited excessive colonization by *C. acnes* accompanied by a reduction in *S. epidermidis*, which may indicate a potential role of follicular dysbiosis in the pathogenesis of the disease.

### 3.9. Itch

An increasing body of evidence indicates that itch associated with skin microbiome dysbiosis results from a complex interplay between bacteria, their metabolites, and the immune and nervous systems [47]. Commensals such as *S. epidermidis*, through the production of enzymes (e.g., sphingomyelinase) and lipid metabolites, support skin barrier function and modulate the local immune environment, which may suppress excessive activation of pruriceptors [9,48]. In turn, metabolites of *C. acnes* (e.g., short-chain fatty acids) have the capacity to limit *S. epidermidis* biofilm formation and attenuate pathogen virulence [49], thereby indirectly reducing itch-inducing stimuli. At the same time, endogenous AMPs such as β-defensins play a dual role, on one hand reinforcing defense against microbes, but on the other acting as pruritogens through activation of Mas-related G-protein coupled receptors (MRGPRs) on sensory neurons [50]. This phenomenon highlights a feedback loop in which the skin’s defensive response to dysbiosis itself exacerbates pruritus. Moreover, disruptions in the TLR–cytokine axis appear central to the persistence of chronic itch. Dysbiosis may amplify TLR3 signaling, promoting the release of pro-inflammatory cytokines and thymic stromal lymphopoietin (TSLP), which enhance Th2 responses and sensory neuron activation [51,52]. Conversely, Gram-positive commensal bacteria via TLR2 may exert protective effects, dampening excessive inflammatory responses [51]. This immuno-neuronal balance between pathogenic and commensal factors is emerging as one of the most important areas of current research on the microbiome and chronic itch. In this context, particularly noteworthy are recent findings by Deng et al. [52], who demonstrated that *S. aureus* and its serine protease V8 can directly induce itch by activating pruriceptors and cleaving protease-activated receptor 1 (PAR-1) on sensory neurons. In murine models, blockade of the SspA–PAR-1 axis alleviated itch and epidermal barrier damage induced by *S. aureus*, pointing to a potential therapeutic target in chronic pruritus [52].

### 3.10. Eczema

Approximately 90% of individuals with eczema exhibit colonization by *S. aureus* along with reduced microbial diversity [53]. Studies also indicate clear differences in the skin microbiome between eczema-prone individuals and healthy controls. In predisposed individuals, characteristic microbiome profiles enriched in potential pathobionts such as *Streptococcus*, *Gemella*, and *Haemophilus* have been observed [6,54].

### 3.11. Carcinogenesis

Microbiome disturbances are associated with skin cancers such as squamous cell carcinoma (SCC), basal cell carcinoma, melanoma, and cutaneous lymphomas [14,55,56] (Table 1). The most common non-melanoma skin cancer (NMSC) is SCC, which frequently develops on the background of actinic keratosis (AK) [55]. During progression from healthy skin (HS) through AK to SCC, a decline in protective commensal *C. acnes* and an increase in pathogenic *S. aureus* have been observed. This shift emerges already at the AK stage but intensifies in SCC, indicating a gradual dysbiosis linked to carcinogenesis. A critical determinant is the *C. acnes*: *S. aureus* ratio, which decreases with lesion progression and may serve as a future biomarker of malignant transformation [55,56]. *C. acnes* produces propionic acid with antimicrobial activity that restricts *S. aureus* colonization [55].

The microbiota of SCC is dominated by microorganisms capable of forming biofilms, inducing inflammation, and evading immune responses. *S. aureus* not only competes with commensals but also activates inflammatory pathways and promotes carcinogenesis [55]. Other microbes, including *P. aeruginosa*, *E. coli*, and *Streptococcus*, can secrete toxic metabolites and genotoxins that cause DNA damage, oxidative stress, and malignant transformation [56]. Additionally, disruptions in tryptophan metabolism (e.g., reduced indole-3-aldehyde) and decreased production of short-chain fatty acids (propionate, valerate) further contribute to inflammation and carcinogenesis [56].

Potential therapeutic strategies include restoring eubiosis through the use of probiotics, microbiota transplantation (particularly in combination with immunotherapy), or modulation of immune responses [56]. *C. acnes* may stimulate anti-tumor immunity via Th1 lymphocyte activation and IFN-γ secretion [55]. Meanwhile, *S. epidermidis* produces 6-HAP (6-N-hydroxyaminopurine), a compound shown to inhibit tumor cell proliferation [22,56].

In melanoma, an increased abundance of potentially pathogenic bacteria such as *Fusobacterium*, *Trueperella*, *Staphylococcus*, *Streptococcus*, and *Bacteroides* has been reported [14,56]. In contrast, elevated levels of *Corynebacterium* have been correlated with more advanced disease stages and higher numbers of IL-17 producing T cells, suggesting a role of this genus in disease progression and amplification of inflammatory responses [57].

**Table 1:** Dermatological disorders associated with the skin microbiota.

Disease	Association with the Skin Microbiome and Consequence	Reference
Acne vulgaris	↑ *C. acnes* (pro-inflammatory strains RT4/RT5), ↓ *S. epidermidis* Consequences: excessive TLR2 stimulation, production of pro-inflammatory cytokines, biofilm formation, inflammation of hair follicles.	[58,59,60,61]
Atopic Dermatitis	↓ diversity, dominance of *S. aureus* (superantigens, toxins), ↓ *S. epidermidis*, ↓ *C. acnes* Consequences: epidermal barrier disruption, enhanced Th2/IgE response, chronic inflammation; excessive *S. epidermidis* growth may also act pathogenically	[29,62,63,64]
Psoriasis	Microbiome changes: ↑ *Streptococcus* spp., ↑ *Malassezia* spp., ↓ *Cutibacterium* Consequences: dysbiosis activation of dendritic cells, predominance of Th17/IL-23 response, exacerbation of skin inflammation.	[65]
Rosacea	Microbiome changes: ↑ *Demodex folliculorum*, ↑ *Heyndrickxia oleronia*, ↓ *C. acnes* Consequences: TLR2 activation, induction of pro-inflammatory cytokines (IL-8, TNF-α), neutrophil recruitment, overexpression of LL-37/KLK5 chronic inflammation, papules, and pustules.	[34,35,66]
Chronic wounds/DFU	Microbiome changes: pathogenic biofilm (*S. aureus*, *P. aeruginosa*), ↓ protective flora; presence of *Porphyromonas*, *Streptococcus*, *Peptostreptococcus*, *Sphingomonas*, *Stenotrophomonas*, *Anaerococcus*, *Staphylococcus*, *Corynebacterium*. Consequences: disruption of the wound-site microbiome balance, chronic inflammation, delayed healing.	[13,17,18]
SD/dandruff	Microbiome changes: ↑ *Malassezia restricta*, ↓ *Malassezia globosa*, ↓ commensal bacteria (*Cutibacterium*, *S. epidermidis*). Consequences: increased production of irritating and pro-inflammatory metabolites (unsaturated fatty acids, indoles), AhR activation, epidermal barrier disruption, increased TEWL, and exacerbation of inflammation.	[39,40,41]
Sebaceous folliculitis	↑ *Malassezia* spp.	[67]
Vitiligo	Microbiome changes: dysbiosis, ↓ microbiome diversity in vitiligo lesions; ↓ *Corynebacterium* (healthy skin), ↑ *Flavobacteriales*, ↑ *Gammaproteobacteria*, ↑ *Flavobacteria*. Consequences: the specific microbiota composition in vitiligo may influence disease progression and the persistence of inflammation.	[42]
Alopecia areata	Microbiome changes: imbalance of the hair follicle microbiome; ↑ *C. acnes*, ↓ *S. epidermidis*; ↓ microbial diversity. Consequences: loss of homeostasis, modulation of immune response, exacerbation of perifollicular inflammation; potential links with the gut microbiome.	[43,44,45,46]
Pruritus/Itch	Microbiome changes: ↓ commensals (*S. epidermidis*, *C. acnes*), ↑ *S. aureus* (proteases, toxins). Consequences: barrier disruption, ↑ pathogenic biofilm, activation of PAR-1/PAR-2 on sensory neurons, overproduction of β-defensins (pruritogenic effect), ↑ TLR3/TSLP signaling -chronic inflammation and persistent itch.	[9,47,48,49,50,51,52,53]
Eczema	Microbiome changes: ↑ *S. aureus*, ↓ microbial diversity; presence of profiles enriched in *Streptococcus*, *Gemella*, *Haemophilus*. Consequences: dysbiosis promotes pathogen colonization, skin barrier impairment, and inflammation development.	[6,54]
AK	Microbiome changes: decrease in *C. acnes* (especially protective strains) and *S. epidermidis*; increase in *S. aureus*. Consequences: dysbiosis appears already in AK and correlates with progression to SCC	[22,55]
SCC	Microbiome changes: ↑ *S. aureus* (biofilm, toxins, inflammation), ↓ commensals (*C. acnes*, *S. epidermidis*). Consequences: dysbiosis promotes progression from AK to SCC; exacerbated inflammation, increased β-defensin-2 expression, impaired immune response.	[55,56,68,69,70]
MM	Microbiome changes: ↑ *Corynebacterium*, *Fusobacterium*, *Streptococcus*, *Trueperella*, *Bacteroides*. Consequences: dysbiosis correlates with disease progression; *Corynebacterium* is associated with an increase in IL-17–producing T cells.	[14,56,57,71]
Cutaneous T-cell lymphoma (CTCL)	Dysbiosis with *S. aureus* dominance (virulent strains, spa/NF-κB) is associated with poorer prognosis ↓ event-free survival. AMPs enhance inflammation and select for pathogens. Commensals (*S. epidermidis*, *S. hominis*) may act protectively. The skin microbiome holds potential as a prognostic marker and therapeutic target.	[72]
BCC	Microbiome changes: decrease in *C. acnes* and *S. epidermidis*; increase in pathogens (*S. aureus*). Consequences: dysbiosis may contribute to BCC development through chronic inflammation and impaired immune response.	[14,55,56]

DFU—diabetic foot ulcers; SD—seborrheic dermatitis; AK—actinic keratosis; SCC—squamous cell carcinoma; MM—malignant melanoma; BCC—basal cell carcinoma, ↑ increase; ↓ decrease.

## 4. Factors Disrupting the Balance of the Skin Microbiome

Lifestyle and skincare routines contribute to the vast diversity of substances that are regularly applied to the skin. These, in turn, influence the microbial diversity across different skin types. Such variability over time depends both on the individual and on the products used [73].

The most important groups of factors that may negatively affect the skin microbiota, along with their mechanisms of action and clinical consequences, are presented below.

### 4.1. Cosmetics

Cosmetic products are intended to improve skin quality and slow down the aging process; however, they may also influence the composition and differentiation of the skin microbiome, particularly with regular or long-term use [74]. Inappropriately selected formulations or their improper application can negatively affect the microbiome by reducing its diversity and leading to dysbiosis [75,76].

Cosmetic ingredients such as carbohydrates, proteins, and lipids may promote the growth of specific skin microorganisms [4]. Lipids contained in moisturizing creams serve as a nutrient source for lipophilic bacteria such as *Cutibacterium* [77,78]. Emulsifiers and preservatives can disrupt the skin’s microbial balance by inhibiting the growth of commensals, including *S. epidermidis* [79,80,81,82]. Parabens, commonly used as preservatives, exert activity against yeasts (*Candida* spp., *Malassezia* spp.) as well as bacteria such as *C. acnes* and *S. epidermidis*. At low concentrations, methylparaben inhibits the growth of yeasts and selected bacteria in vitro. Methylisothiazolinone has been shown to suppress *S. epidermidis*, while phenoxyethanol may disrupt the skin microbiota by increasing the abundance of *Proteobacteria* and decreasing *S. epidermidis* [82,83,84]. The effects of preservatives strongly depend on concentration, duration of exposure, and the individual resilience of the microbiome [85].

Gelling and thickening agents have not demonstrated adverse effects on the growth of *S. epidermidis* under in vitro conditions [86], making them microbiome-friendly.

Skincare products, particularly cleansers, often have an alkaline pH. Deviation from the skin’s natural acidic environment disturbs its microbiome, leading to changes in microbial populations and, in some cases, dermatological disorders [87]. Elevated pH favors the proliferation of *C. acnes* and *S. aureus* [88], as well as the transformation of commensal *C. albicans* into its pathogenic form [89].

### 4.2. Surfactants

Surfactants, particularly those with strong activity, can inhibit microbial growth, while metabolites generated through their degradation by the skin microbiota further alter the cutaneous environment [90]. Harsh cleansing agents, especially anionic surfactants such as sodium lauryl sulfate (SLS), widely used in cosmetics and dermatological formulations, dissolve lipids, denature proteins, and remove microorganisms from the skin surface. Excessive use of soap disrupts the skin microbiome, reducing its diversity by damaging the epidermal surface and weakening its protective functions [4,54]. Detergents non-selectively eliminate commensal microbiota (e.g., *S. epidermidis*, *C. acnes*), resulting in microbial imbalance [17]. Skin model studies have demonstrated that cleansing with SLS-containing formulations significantly reduces the abundance of certain commensals even within a short period, with the effect depending on concentration, exposure time, and sampling site. Clinically, this manifests as skin dryness, exacerbation of AD, pathogen colonization (e.g., *S. aureus*), and irritation [4]. Milder alternatives include non-ionic surfactants such as glucosides, as well as synthetic detergents (syndets) with neutral pH.

### 4.3. Microplastics

An increasing body of evidence suggests that microplastics present in cosmetics (e.g., polyethylene, polyethylene terephthalate, polypropylene) may not only exert mechanical effects but also impact the skin microbiome. These particles can adsorb environmental contaminants and promote biofilm formation, thereby potentially facilitating pathogen transfer and disrupting the microbial balance of the skin [83]. Experimental data indicate that contact of micro- and nanoplastics with the skin triggers an immune response comparable to that induced by pathogens. The particles are recognized by keratinocyte and Langerhans cell receptors, leading to the release of AMPs and pro-inflammatory cytokines (IL-1, IL-6, IL-17, TNF), which may disturb the balance between commensal and pathogenic microorganisms [91]. It has also been demonstrated that microplastics can bind proteins, altering their interactions with cells, and provide a surface favorable to bacterial colonization and biofilm formation [92]. Furthermore, microplastics may induce oxidative stress and premature skin cell aging, which affect lipid metabolism and weaken the hydrolipid barrier, thereby promoting dysbiosis [93].

These mechanisms suggest that microplastics may not only exert irritating and cytotoxic effects but also actively modify the skin’s microbial environment, increasing the risk of chronic inflammatory conditions.

### 4.4. Antibiotics

The use of antibiotics in the treatment of skin diseases is a standard procedure. However, both topical and systemic antibiotic administration may reduce microbiome diversity, select for resistant strains, and in some cases facilitate colonization by *S. aureus*. Appropriate antibiotic selection determines therapeutic efficacy while minimizing collateral effects on other microorganisms inhabiting the skin surface [4].

### 4.5. Dermatological Procedures

Dermatological procedures can also have a destructive impact on the skin microbiome [94]. Broad-spectrum bactericidal interventions, such as laser therapy or high-frequency electrotherapy, may eliminate both pathogenic and commensal microorganisms [95,96]. Light-based therapy used in acne treatment has in some cases been associated with an increased abundance of staphylococci, including potentially pathogenic strains, which may promote the development of inflammatory skin conditions such as AD [97]. Chemical peels, particularly those with high concentrations of active substances (e.g., salicylic acid), can reduce the populations of *S. epidermidis* and *C. acnes* in otherwise healthy skin areas, thereby disturbing microbial homeostasis [98,99]. Removal of the epidermal layer or deeper skin structures by mechanical procedures such as dermabrasion or surgical debridement may cause a temporary loss of protective biofilms and commensals. Despite these potential risks, dermatological procedures may also beneficially modulate the skin microbiome. Narrowband UVB phototherapy, photodynamic therapy (ALA-PDT), and selected chemical peels used in conditions such as AD, psoriasis, or acne have been shown to reduce the abundance of pathogens (*S. aureus*, *C. acnes*) and increase microbial diversity, thereby restoring a profile more closely resembling that of healthy skin [94,98].

### 4.6. Ultraviolet (UV) Radiation

UV radiation has been shown to affect the composition and activity of the skin microbiota, with consequences that may be either beneficial, such as reducing the abundance of opportunistic pathogens like *S. aureus*, or detrimental, for example, by promoting chronic inflammation [100]. Commensal and protective microorganisms such as Lactobacillus and *C. acnes* are particularly sensitive to UV exposure [101,102]. A reduction in these bacteria can disrupt microbiome homeostasis and weaken protective functions. For instance, decreases in Lactobacillaceae and Pseudomonadaceae populations, along with an increased relative abundance of *Cyanobacteria*, have been reported following UV exposure [101,102]. Even microorganisms with certain protective mechanisms, such as *Malassezia furfur*, which synthesizes pityriacitrin (a natural UV filter), demonstrate high sensitivity to UV radiation [102]. Harel et al. [103] investigated the effects of solar radiation on the skin microbiome of lifeguards chronically exposed to high UV doses and ultra-Orthodox Jews who were protected year-round by heavy clothing. They found that sun exposure induced changes in microbiome composition and diversity, including reductions in the abundance of specific bacterial species. These findings indicate that solar UV radiation may negatively impact skin microbiome health by reducing beneficial bacteria and increasing the proportion of potentially pathogenic species.

### 4.7. Air Pollution

Air pollutants such as ground-level ozone (O_3_), particulate matter (PM), and gaseous contaminants including sulfur dioxide (SO_2_), lead, carbon monoxide (CO), nitric oxide (NO), nitrogen dioxide (NO_2_), and nitrous oxide (N_2_O) [104] exert a significant negative impact on the biodiversity of the skin surface and the composition of its microbiota [105,106]. A study by Chueachavalit C. et al. [106] demonstrated that high concentrations of air pollutants influence skin colonization by *Malassezia* spp., which may lead to dysbiosis of the skin ecosystem and consequently contribute to the development of certain dermatological diseases [106].

### 4.8. Place of Residence

Significant differences in skin microbiome composition are observed between individuals living in rural and urban environments. Greater intra-group structural variability has been reported among rural inhabitants, whereas urban residents demonstrate higher relative abundance of Trabulsiella on the hands, forearms, and forehead, as well as a greater abundance of *Cutibacterium* in women. In contrast, *Corynebacterium* predominated in women from rural areas [107]. Chemical analyses have revealed higher concentrations of detergents, cleaning agents, and pharmaceutical residues in urban households. A negative correlation between detergent use and microbiome diversity has also been demonstrated [108]. With urbanization, the proportion of potentially pathogenic taxa (both bacterial and fungal) increases, which may explain the higher prevalence of diseases such as acne, AD, and *S. aureus* infections. Cordain L. et al. [109] reported that acne is absent among indigenous populations but emerges following relocation to cities. Given projections that by 2050 as much as two-thirds of the global population will live in urban areas [110], supporting a healthy human and indoor microbiota becomes critical to mitigating the worldwide rise in civilization-associated diseases. Indoor climatic conditions are also of importance. Environmental factors such as low humidity and reduced temperature limit sebaceous gland activity, leading to decreased sebum production—an essential lipid source for lipophilic bacteria, including *C. acnes*. Fournière et al. [79] emphasized the role of these bacteria in maintaining skin microbiome homeostasis and their sensitivity to changes in the epidermal microenvironment. Additional data from Frontiers in Microbiology [111] showed that dry skin areas in young women, characterized by reduced hydration, TEWL, and sebum secretion, had significantly lower relative abundance of *Actinobacteriota*, including *Cutibacterium*. Furthermore, contrary to earlier assumptions, more precise insights into correlations between environmental conditions and the skin microbiome were provided by a longitudinal facial skin study in adults [112], which demonstrated seasonal shifts in microbiome composition including *Cutibacterium* associated with biometric skin parameters.

### 4.9. Excessive Hygiene

Excessive hygiene, including frequent use of detergents and antibacterial agents, may lead to imbalances in the skin microbiome. Strong surfactants with alkaline pH remove natural lipids and components of the natural moisturizing factor (NMF), resulting in epidermal barrier damage, reduced diversity of commensal microbiota, and increased susceptibility to pathogen colonization. This phenomenon is associated with the exacerbation of inflammatory skin diseases such as AD and acne [113,114]. There is a growing emphasis on the need to redefine the concept of hygiene, with the goal of reducing pathogens while maintaining microbiome balance, rather than eliminating all microorganisms from the skin [115]. Overuse of antiseptic and antibiotic agents further promotes dysbiosis and the selection of resistant strains, with clinical consequences not only for skin health but also for the host’s overall immunity [13].

### 4.10. Age and Skin Aging

With age, decreased sebum production, reduced hydration, increased TEWL, elevated skin pH, and gradual flattening of the dermo-epidermal junction are observed. These changes limit the exchange of nutrients and oxygen and weaken skin barrier integrity, thereby affecting host–microbe mutualistic interactions [116]. Aging is also accompanied by a decline in commensal bacteria such as *Cutibacterium* spp. and *Lactobacillus* spp., which produce glycerol, free fatty acids, and other protective metabolites. As a result, protection against pathogen colonization is reduced, promoting dysbiosis [117,118]. Although elderly individuals may exhibit greater microbiome diversity, this occurs at the expense of ecosystem stability and the abundance of beneficial species, ultimately weakening the skin’s defense mechanisms [119]. In addition, collagen synthesis, sebum production, and antimicrobial peptide (AMP) activity decline with age, increasing susceptibility to infections [120].

### 4.11. Chronic Stress

Chronic psychological stress activates the hypothalamic–pituitary–adrenal (HPA) axis, resulting in prolonged elevation of cortisol levels. Cortisol reduces the activity of macrophages and lymphocytes and disrupts epidermal immune functions [121]. At the same time, the skin microbiota plays a key role in maintaining local immune homeostasis by regulating inflammatory responses and T-cell activity. This indicates that stress, through its impact on the immune system, indirectly affects the interactions between the skin and its microbiome, thereby promoting potential imbalances [122].

## 5. Strategies for Microbiome-Friendly Intervention

Strategies to correct dysbiosis include (i) probiotics (live microbes), (ii) prebiotics (non-digestible nutrients for commensals), (iii) postbiotics (microbial metabolites or lysates), and (iv) microbiome-compatible cosmeceuticals (botanical extracts, biosurfactants, etc.). We organize the evidence for each, emphasizing mechanisms.

### 5.1. Probiotics

Probiotics are live microorganisms that confer health benefits when applied in adequate amounts [123,124,125]. In dermatology, topical probiotics (often *Lactobacillus* or *Bifidobacterium* strains) are being explored to restore skin barrier and microbial balance. Probiotics act by multiple mechanisms: they competitively exclude pathogens for niches and nutrients, produce antimicrobial compounds (bacteriocins, organic acids), and modulate host immunity [126]. For instance, certain *Lactiplantibacillus* strains secrete lactic acid (lowering pH), bacteriocins that disrupt pathogen membranes, and can interfere with pathogen quorum sensing [127]. Probiotics also stimulate keratinocyte and immune signaling: by interacting with Toll-like receptors (TLRs), they can increase regulatory cytokines (e.g., IL-10) and expand T-regulatory cells, thereby dampening inflammation [127].

Clinical studies have shown benefits of topical probiotics in AD and acne. For example, a cream with heat-killed *Streptococcus thermophilus* increased ceramide levels and eased AD symptoms [128]. Topical extracts of *L. sakei* and *L. johnsonii* have reduced *S. aureus* colonization in AD, supporting barrier repair [129,130]. Moreover, an ointment containing the live strain *Lactobacillus reuteri* reduced AD severity after four and eight weeks of therapy [131]. A beneficial effect was also observed with the topical application of the commensal bacterium, which was associated with reduced *S. aureus* colonization and a lower need for corticosteroids [132].

In acne therapy, promising results were reported for a preparation containing live strains of *Lacticaseibacillus rhamnosus* GG, *Lactobacillus plantarum* WCFS1, and *Lactiplantibacillus pentosus* KCA1, which modulated the skin microbiota and decreased the abundance of *C. acnes* and staphylococci [133]. Another product, SkinDuo™ (live *Lactiplantibacillus plantarum*), lowered inflammatory mediators and decreased pathogenic *C. acnes* while promoting commensals like *Rothia mucilaginosa* [134].

These outcomes likely reflect the mechanisms above. Importantly, probiotic effects are strain-specific and depend on viability [135]. Different strains of *L. plantarum*, for example, may vary in bacteriocin production or immunomodulatory potency. Maintaining live organisms in formulations is technically challenging; stable delivery systems (creams, gels, serums) are required to preserve viability through shelf life [135]. Formulation factors—dose, vehicle pH, and use of preservatives—also affect probiotic activity.

In summary, probiotic therapies can competitively inhibit pathogens (via organic acids and AMPs), strengthen the skin barrier (enhancing ceramides, filaggrin, tight junctions), and modulate immunity (e.g., ↑ IL-10, ↓ pro-inflammatory Th17 responses). Their safety profile tends to be good, but strain identity and purity must be specified.

### 5.2. Prebiotics

Prebiotics are substrates selectively utilized by host microbes to confer a health benefit [136]. Topical prebiotics (e.g., oligosaccharides, β-glucans, inulin) nourish beneficial skin commensals. By supplying fermentable carbon sources, they encourage production of lactic acid and short-chain fatty acids (SCFAs) by resident bacteria. These fermentation byproducts lower the skin pH and inhibit pathogens. For example, in vitro fermentation of inulin-derived fructooligosaccharides by *S. epidermidis* produces lactic acid that suppresses *S. aureus* biofilm formation. Likewise, β-glucans from oats stimulate *S. epidermidis* growth and metabolism, leading to higher lactic acid levels. Lower pH and the presence of antimicrobial metabolites make the environment hostile to opportunists, favoring commensal stability [137,138].

Prebiotics also indirectly modulate the immune system. Commensals fermenting prebiotics may produce metabolites that induce host anti-inflammatory pathways [139]. For example, microbial utilization of certain oligosaccharides can enhance regulatory cytokine production. Clinical and in vitro studies support prebiotic benefits: topical β-glucan and galacto-oligosaccharide formulations have improved dry skin and atopic symptoms by normalizing microbial communities. A clinical trial of 1% colloidal oatmeal (β-glucan source) cream found increased *S. epidermidis* growth, reduced *Staphylococcus* spp. on lesions, and improvements in barrier function and hydration compared to control [140]. These findings suggest that prebiotics can selectively feed commensals, maintain a healthy diversity, and reinforce the skin’s protective chemistry.

#### 5.2.1. Oligosaccharides

In recent years, natural prebiotic oligosaccharides have attracted increasing interest in dermatology and cosmetology due to their ability to selectively promote the growth of beneficial commensal skin bacteria while simultaneously limiting the development of pathogens [136,141]. Prebiotic oligosaccharides support the skin microbiome by providing a selective carbon source for commensals, which in turn produce metabolites such as lactic acid and short-chain fatty acids (SCFAs). These compounds lower the skin’s pH and hinder pathogen proliferation [141].

Oligosaccharides may also indirectly strengthen microbial interactions by supplying metabolites to other bacteria and by stimulating the secretion of antimicrobial substances, while simultaneously activating the host’s immune response [142]. Both clinical and preclinical studies confirm that topical and oral administration of oligosaccharides improves skin condition including in AD acne vulgaris, skin dryness, and in the prevention of photoaging [141].

#### 5.2.2. Algal Oligosaccharides

Marine algae, due to their high content of polysaccharides and oligosaccharides, also demonstrate prebiotic potential [143]. Recent studies have shown that cosmetic ingredients containing algal extracts can restore a healthy skin microbiome and increase microbial diversity after stress-induced dysbiosis [144].

Fournière et al. [145] investigated oligosaccharides obtained from *Ulva* sp. (green algae, *Chlorophyta*, *Ulvales*, *Ulvaceae*). Variants of these oligosaccharides did not significantly alter the cytotoxicity of *S. aureus* and *S. epidermidis* toward HaCaT keratinocytes in vitro, but they modified the structure of bacterial biofilms and reduced by 40% the pro-inflammatory potential of both acne-associated and non-acne-associated *C. acnes* strains [145].

It has also been demonstrated that a complex of extracts derived from brown algae (*Laminaria digitata*), green microalgae (*Chlorella vulgaris*), marine exopolysaccharides, and seawater increases microbial diversity. The bioactive compounds present in this complex promote bacterial proliferation by providing essential nutrients and protecting against drying stress [144].

#### 5.2.3. Pectic and Starch Oligosaccharides

Pectic and starch oligosaccharides derived from banana peels (*Musa* spp.) exhibit prebiotic properties toward the skin microbiome. In in vitro studies, their effects on the growth of the commensal *S. epidermidis* and the opportunistic *C. acnes* were evaluated. Both fractions significantly stimulated the proliferation of *S. epidermidis*, while the pectic fraction additionally exerted a strong inhibitory effect on the growth of *C. acnes*, suggesting a potential role in the selective modulation of the skin microbiota [146].

#### 5.2.4. Chitosan Oligosaccharides

Chitosan oligosaccharides (COS) are short chains of glucosamine obtained from crustacean shells (e.g., shrimp, crabs), fungi, or other sources of chitin, using chemical or enzymatic methods. They support the growth of selected commensal skin bacteria, modulate the microbial environment, and exhibit antimicrobial activity. Studies have shown that oligochitosan with a molecular weight of 10 kDa demonstrates the highest efficacy against *C. acnes*. In the context of skin care and the treatment of dermatoses associated with microbiome dysbiosis (e.g., acne vulgaris), COS may therefore play a dual role: on the one hand, supporting beneficial microorganisms as a mild prebiotic, and on the other, limiting pathogens (such as *C. acnes*) and enhancing the effectiveness of conventional antibacterial therapies. This dual activity makes COS a promising ingredient in dermocosmetics and dermatological adjuvant formulations [147].

#### 5.2.5. Milk Oligosaccharides

The best-known oligosaccharides include human milk oligosaccharides (HMOs) [148] and galactooligosaccharides (GOS), which are produced from lactose [149]. Studies by Mortaz et al. [150] demonstrated that GOS can directly inhibit the growth of *S. aureus* and *P. aeruginosa*, while also enhancing neutrophil phagocytic activity against these pathogens, suggesting their therapeutic potential in wound healing. GOS exhibit selectivity toward specific microorganisms, thereby supporting commensals while limiting pathogens. In vitro experiments showed that GOS at concentrations above 2.5% significantly suppressed the growth of *S. aureus* and *P. aeruginosa* [150].

#### 5.2.6. Inulin (Fructooligosaccharides) (FOS)

Inulin is a prebiotic ingredient widely used in cosmetics for its ability to support the growth of beneficial bacteria as well as for its moisturizing properties [151]. It is a naturally occurring storage carbohydrate found in leeks, onions, wheat, asparagus (*Asparagus officinalis*), garlic, Jerusalem artichoke (*Helianthus tuberosus*), and chicory root (*Cichorium intybus*). In industrial production, two members of the Asteraceae family are primarily used as sources of inulin: Jerusalem artichoke and chicory, with chicory being the most commonly utilized [152].

FOS, obtained from inulin through enzymatic hydrolysis, also demonstrate skin microbiome-supporting activity [153]. Shao et al. [141] showed that FOS at a 2% concentration significantly stimulated the growth of the commensal *S. epidermidis*, while their fermentation products inhibited *S. aureus* biofilm formation.

#### 5.2.7. Gluco-Oligosaccharides (GlcOs)

GlcOS, which include isomaltooligosaccharides (IMOs) and gentioligosaccharides (GnOS), are produced from corn, tapioca, or from sucrose/maltose through enzymatic processes [154]. In vitro studies have shown that they are extensively metabolized by commensal skin bacteria such as *Rothia kristinae*, *Kytococcus sedentarius*, *Staphylococcus capitis*, *Corynebacterium xerosis*, and *Lactiplantibacillus pentosus*, whereas pathogens including *S. aureus*, *Gardnerella vaginalis*, and *C. acnes* do not exhibit this capacity [154]. Importantly, fermentation of GlcOS by commensals creates an acidic microenvironment that suppresses pathogen growth, while GlcOS themselves can additionally reduce glycocalyx production by *S. aureus*, thereby impairing its ability to form biofilms [155].

#### 5.2.8. Oat (*Avena sativa* L.)—Beta-Glucan

Colloidal oat (*Avena sativa* L.) has been used topically for centuries in the treatment of skin disorders such as AD and other conditions associated with dryness and impaired barrier function [156,157]. In addition to starch, proteins, lipids, and fiber, colloidal oat contains approximately 5% β-glucans, which serve as a nutritional substrate [156]. According to studies by Liu-Walsh et al. [158], β-glucan significantly stimulates the growth and metabolism of *S. epidermidis*, simultaneously increasing the concentration of lactic acid, a natural moisturizing factor in the stratum corneum. It has been demonstrated that the use of a moisturizing cream containing 1% colloidal oat enhances the growth rate of *S. epidermidis* [158]. Treatment with a 1% colloidal oat cream, in contrast to a standard emollient, reduced the presence of *Staphylococcus* species, increased microbiome diversity at lesion sites, and significantly improved skin pH, barrier function, and hydration. These findings confirm that such a cream beneficially modulates the microbiome and strongly supports skin barrier regeneration [159].

Furthermore, extracts from young oat shoots, rich in saponins and flavonoids, exhibit anti-inflammatory properties, stimulate keratinocyte proliferation, and enhance the production of hyaluronic acid, type IV collagen, sphingomyelin, ceramides/cerebrosides, and free fatty acids. Clinically, this translates into improvements in symptoms such as pruritus and skin dryness [160].

### 5.3. Postbiotics

Postbiotics are inanimate microbial preparations or their components that confer health benefits [161]. Many cosmetic “fermented” extracts fall into this category [162]. Unlike live probiotics, postbiotics deliver microbial metabolites, cell wall fragments, or lysates that carry activity. Common postbiotics from *Lactobacillus* spp. and *Bifidobacterium* include enzymes, peptidoglycans, exopolysaccharides, and teichoic acids. These compounds have diverse actions: they can modulate host immunity, reduce inflammation, act as antioxidants, and strengthen the skin barrier [161,162,163,164]. For instance, lysates of *L. rhamnosus* GG and *B. longum* have been shown in vitro to enhance tight-junction proteins and barrier resilience [164]. Topical application of *L. plantarum* lysate creams has reduced acne lesions in clinical studies, likely by inhibiting *C. acnes* and soothing inflammation [165,166]. Postbiotics derived from *L. fermentum* and *L. reuteri* have accelerated wound healing in animal models by promoting keratinocyte migration and reducing cytokines [167]. These benefits suggest that even without live cells, microbial components can confer competitive inhibition of pathogens (via contained AMPs or acids) and immunomodulation of the skin. Studies have also confirmed the antioxidant properties of postbiotics, showing that they protect against UVB-induced skin damage, limit melanin production, and support the function of the epidermal barrier [168].

#### 5.3.1. Fermented Oils

Fermented oils are plant-derived oils subjected to a biotechnological process involving the yeast *Pseudozyma* sp. SY-16. This process enhances their antioxidant, anti-inflammatory, and antibacterial properties, while also enabling them to act as postbiotics by influencing the composition and activity of the skin microbiome. In a clinical study conducted by Ciardiello et al. [169], three such oils were evaluated: F-Shiunko^®^—a mixture of apricot kernel oil, olive oil, sweet almond oil, and sunflower oil, enriched with extracts of Angelica gigas and Lithospermum erythrorhizon, traditionally used for wound healing, AD, and eczema; F-Artemisia^®^—an oil containing extract of *Artemisia* princeps, known for its soothing, anti-inflammatory, and barrier-restoring effects; F-Glycyrrhiza^®^—an oil with *Glycyrrhiza glabra* (licorice root) extract, rich in glycyrrhizin, glabridin, and isoflavones with strong anti-inflammatory activity. After four weeks of applying emulsions containing these oils to the cheek skin of healthy female volunteers, a significant increase in alpha-diversity of the skin microbiota was observed, along with a reduction in *Proteobacteria* and *C. acnes*, and an increase in commensal *Staphylococcus* species (including *S. epidermidis*). The resulting microbiological profile was considered beneficial for maintaining microbiome balance and skin immunity. These findings suggest that fermented oils may effectively support skin microbiome homeostasis and improve overall skin condition [169].

#### 5.3.2. Fermented Sugarcane Straw (*Saccharum officinarum* L.)

Fermented sugarcane straw (*Saccharum officinarum* L.) represents a novel, sustainable source of postbiotics obtained through fermentation with *Saccharomyces cerevisiae*. Duarte et al. [162,163] evaluated its effects on the skin microbiome in an in vivo study involving nine female volunteers. Analysis of the relative abundance of selected genera and species revealed that the extract significantly reduced the proportion of *C. acnes* and *Malassezia* yeasts, while having no notable impact on the presence of *S. aureus*, *S. epidermidis*, *Corynebacterium* spp., or *P. innocua*. Importantly, although an overall increase in the total fungal population on the skin was observed, the proportion of pathogenic *Malassezia* decreased. These findings suggest that the postbiotic derived from fermented sugarcane straw may modulate the skin microbiome by reducing pathogenic microorganisms while maintaining the balance of commensal bacteria. In addition, the extract demonstrated antioxidant, anti-inflammatory, elastase-inhibitory, and tyrosinase-inhibitory activities [162,163].

Indeed, postbiotics essentially combine prebiotic and direct bioactivity: they may contain organic acids (like lactic acid) and SCFAs from prior fermentation, which can lower pH and suppress pathogens, while also delivering microbial surface molecules that engage skin immune receptors. For example, certain bacterial polysaccharides act as antioxidant humectants, and peptides may serve as signaling molecules to boost IL-10 or barrier lipids. This is reflected in the mechanistic summary (Table 2): postbiotics are reported to provide anti-inflammatory/immunomodulatory effects, strengthen the epidermal barrier, reduce pathogen colonization, and improve hydration. As with probiotics, safety and efficacy depend on the strain source and processing. Fermented ingredients (e.g., kefir, kombucha) may vary widely in composition. Thus, explicit identification of microbial source and quality control are necessary.

In the context of cosmetic microbiome ingredients, safety hinges on the profile of adverse effects such as irritation/allergy and the resistome/antimicrobial resistance, and must also consider the viability of declared probiotics in leave-on products and the horizon of long-term use [170]. Products claiming probiotics/postbiotics should include a Cosmetic Product Safety Report with microbiological quality data; strain-level identification for any probiotic; or, for postbiotics, documentation of the source microorganism and inactivation process. A commercial product containing live probiotics should provide an adequate quantity of viable cells and have verified stability/viability throughout its entire shelf life [171]. At the same time, the primary risk specific to live probiotics remains antimicrobial resistance: prior to implementation, Minimum Inhibitory Concentration (MIC) testing against cut-off values and genomic analyses (e.g., CARD, the Comprehensive Antibiotic Resistance Database, and ARG-ANNOT, Antibiotic Resistance Gene-ANNOTation) should be performed to detect mobile resistance elements. Strains with potentially transferable resistance must be excluded—particularly relevant for long-term, daily use of leave-on products [170]. From a formulation perspective, probiotic viability is sensitive to water activity, oxygen, pH, format, and packaging; therefore, viability and metabolic activity must be validated at the end of shelf life [170]. Postbiotics meet identifiability requirements without the need to maintain viability, which simplifies microbiological quality control in leave-on formats and reduces infectious risk. Despite encouraging short-term findings, the literature emphasizes the lack of long-term data and the need for longer RCTs to fully characterize safety (including potential endocrine effects, which to date have not been reported as a concern in cosmetic studies) [171].

### 5.4. Microbiome-Friendly Cosmeceuticals

Beyond classical pre-/pro-/post-biotics, various cosmetic ingredients support the microbiome. Botanical extracts (rich in polyphenols, terpenes, etc.) often have dual action: they inhibit pathogens while sparing commensals. For instance, green tea (*Camellia sinensis*) leaf extract selectively enhances dendritic cell signaling to improve pathogen clearance without harming beneficial skin bacteria. Witch hazel (*Hamamelis virginiana*) extract inhibited *S. aureus* and *E. faecalis* but supported *L. plantarum* in culture. Pomegranate extract (*Punica granatum*) taken orally increased skin *S. epidermidis* and *Bacillus* populations and improved barrier function in humans, likely via its antioxidant polyphenols. Some oils (e.g., fermented plant oils) have been shown in trials to increase overall diversity and commensals while reducing potential pathogens (e.g., *Malassezia*, certain *Corynebacteria*).

It should be emphasized that modern cosmetology is increasingly oriented toward the use of natural, minimally processed plant-derived ingredients that support the balance between the host and its microbiome. It is increasingly emphasized that maintaining skin microbiota homeostasis is a key element of skin health prevention, highlighting the need to develop formulations that foster symbiosis with commensal microorganisms while minimizing the risk of dysbiosis [85,172]. According to the concept of so-called “microbiome-compatible formulations,” skincare should be personalized to the individual microbiome profile. In practice, this involves the use of standardized or raw plant extracts that support the microbiota by maintaining populations of beneficial bacteria such as *S. epidermidis* and by modulating the skin environment without excessive interference in its natural ecology. Such an approach not only improves the appearance of the skin but also strengthens its defensive functions and supports overall well-being.

Interactions between microorganisms and plants can be considered in two ways. On the one hand, medicinal plants rich in bioactive compounds influence microbial growth and metabolism, acting against bacteria, fungi, and viruses. On the other hand, metabolically active microbial communities may modify the chemical composition and structure of plant-derived preparations applied to the skin. Numerous natural substances show documented antimicrobial properties: phenolics exhibit antifungal and antiviral effects, quinones inactivate bacterial proteins, and tannins disrupt enzymes and adhesion proteins [173,174,175,176]. Both in vitro and in vivo studies also confirm the effectiveness of certain plant preparations against skin pathogens. *Chamomille* essential oil and bisabolol inhibit the growth of Gram-positive bacteria, including *S. aureus* and *Bacillus subtilis*, as well as the fungus *C. albicans*. Moreover, aqueous extracts of onion (*Allium cepa*) demonstrate antifungal activity against *Malassezia furfur*, various *Candida* species, and dermatophytes [174].

Compounds originating from the skin microbiome have significant therapeutic and cosmetic potential. They may be applied in the treatment of skin cancers and antibiotic-resistant infections, as well as in formulations designed to alleviate acne, eczema, and signs of skin aging by modulating both skin properties and its microbiome [177]. Consequently, there is a growing emphasis on developing cosmetic formulations that minimize the risk of disturbing the natural microbial balance of the skin. This trend has led to increasing interest within the cosmetic and dermatological industries in creating so-called “microbiome-friendly” or “microbiota-safe” products [178]. Microbiome-supportive skincare can significantly enhance skin microbial diversity, improve skin structure, and reduce redness compared with conventional products [179]. To achieve these effects, ingredients that strengthen the epidermal barrier and help maintain natural skin pH are commonly used [93]. These include bioactive compounds of plant, algal, or thermal water origin, which do not serve as nutrient sources for microorganisms; animal-derived ingredients and minerals, which likewise do not feed commensals but instead support their survival [79]; prebiotics and postbiotic [93,180,181,182]; probiotics [93].

The key applications of microbiome-friendly cosmetic active ingredients include: promoting the metabolism of commensal bacteria and/or enhancing microbial diversity while maintaining an appropriate *S. epidermidis*/*C. acnes* ratio to limit pathogen invasion; reducing the growth, virulence, and biofilm formation of pathogens; and modulating the skin microenvironment and immune responses [79].

#### 5.4.1. Botanical Extracts

##### *Rhodomyrtus tomentosa* (Aiton) Hassk.

Studies have shown that an extract from *Rhodomyrtus tomentosa* fruit, applied at a concentration of 2%, modulated the skin microbiota by altering the proportions of specific *C. acnes* phylotypes. An increase in the abundance of phylotypes II and III was observed, along with a simultaneous decrease in phylotype IA1 [183]. It is well established that acne is associated with reduced *C. acnes* phylotype diversity and dominance of phylotype IA1. Therefore, a strategy aimed at supporting phylotype diversity within this species—by limiting IA1 abundance while increasing the representation of other phylotypes—appears to hold significant preventive and therapeutic potential.

##### *Halymenia* *durvillei*

*Halymenia durvillei* is a red alga from the family Rhodophyceae, naturally occurring in the Indian Ocean. Its main bioactive components are phycocolloids—polysaccharides that form part of the cell membranes. Growing interest in these compounds arises from their demonstrated biological activity, including immunomodulatory properties and protective effects on the skin. In a clinical study conducted by Filaire et al. [184] in patients with reactive and sensitive skin, a 28-day application of a formulation containing red algae extract resulted in a significant reduction in *Corynebacterium kroppenstedtii*, a bacterium associated with skin redness and hyperreactivity, along with a concomitant increase in *S. epidermidis*, known for its beneficial role in maintaining skin homeostasis and health. Importantly, the preparation also prevented a decline in skin microbial diversity, thereby supporting the balance of the skin microbiome ecosystem [184].

##### *Mangifera indica* L.

*Mangifera indica* is a plant from the *Anacardiaceae* family, whose leaves are a rich source of phenolic compounds such as mangiferin, iriflophenone, and maclurin, which exhibit anti-inflammatory, antioxidant, and sebum-regulating properties. The ethanol extract of *M. indica* leaves represents a potential anti-acne ingredient with multifaceted activity—ranging from modulation of lipogenesis to effects on the skin microbiome. Studies have shown that the extract reduces lipid production in sebocytes and decreases *C. acnes* lipase activity, thereby limiting the excessive release of free fatty acids responsible for inflammation within the pilosebaceous unit. Of particular importance is its effect on the skin microbiota. In clinical trials, the extract maintained a stable microbial composition, preventing dysbiosis-like changes observed in the placebo group. *M. indica* preserved commensal bacterial populations such as *S. epidermidis*, while not inducing overgrowth of potential pathogens such as *Acinetobacter* or *Lawsonella*. Through this mechanism, the extract supports the maintenance of a balanced skin microbiota, contributing to skin regeneration and the prevention of dermatoses [185].

##### *Symphytum officinale* L.

The hydroethanolic extract of *Symphytum officinale* L. root has been shown to exert beneficial effects on the skin microbiome. Ex vivo studies confirmed that it does not induce dysbiosis or reduce alpha-diversity but instead supports microbial stability through subtle qualitative changes in community composition. Skin microorganisms are capable of actively metabolizing certain compounds present in the extract (e.g., polyphenols, allantoin), leading to the formation of metabolites with potential anti-inflammatory and regenerative activity. These findings suggest that the skin microbiome participates in the biotransformation of extract components in a manner that may enhance its therapeutic effect, support microbial balance, and strengthen the skin’s defensive functions [186].

##### *Calendula officinalis* L. and *Arnica montana* L.

Studies have shown that the hydroethanolic extract of *Calendula officinalis* flowers inhibits the growth of *C. acnes* and may also reduce the proliferation of pathogenic *S. epidermidis*. However, this species also plays important protective roles within the skin microbiome [187]. In broader in vitro studies, aqueous and ethanolic flower extracts of marigold inhibited bacterial growth at concentrations ranging from 125 µg/mL to 64 mg/mL, with *S. aureus* being the most susceptible. The methanolic flower extract was also active against *S. aureus* at 64 mg/mL [188]. Further experiments confirmed the activity of aqueous, ethanolic, chloroform, and ether extracts from the leaves against *Bacillus subtilis* and *S. aureus*, as well as against the fungi *C. albicans* and *Aspergillus niger* [189]. In another study, ethanolic and methanolic extracts exhibited inhibitory effects against *C. albicans* [190]. These findings suggest that calendula may support acne therapy by modulating the skin microbiome and reducing the abundance of pathogenic bacteria. In the case of mountain arnica (*Arnica montana*), there are reports of its beneficial effects on the composition of the skin microbiome; however, fundamental experimental studies that would unequivocally confirm these observations are still lacking. Therefore, the need for further research in this direction is emphasized [186].

##### *Centella asiatica* (L.) Urb.

Studies by Bikiaris et al. [191] on a serum containing *Centella asiatica* extract (CAE) confirmed its broad skin-care and protective effects. The preparation demonstrated significant antibacterial activity, particularly against *S. aureus*, indicating the potential for indirect modulation of the skin microbiome through pathogen reduction while preserving commensals. In addition, the extract exhibited antioxidant, anti-inflammatory, anti-tyrosinase, and skin barrier–strengthening properties, all of which contribute to maintaining microbiological homeostasis and overall skin health.

##### *Hamamelis virginiana* L.

Studies by Rasooly et al. [192] demonstrated that a commercial ethanol–water extract of *Hamamelis virginiana* bark exerts selective effects on skin microorganisms. The extract inhibited the growth of pathogenic bacteria, reduced biofilm formation and toxin production, with particularly strong effects against *S. aureus*, *S. epidermidis*, and *Enterococcus faecalis*. At the same time, it supported the growth of the probiotic bacterium *Lactobacillus* plantarum under both nutrient-poor and nutrient-rich conditions, and further protected it from oxidative stress. These findings highlight the potential of witch hazel (*H. virginiana*) in modulating and maintaining a healthy skin microbiome [192].

##### *Camellia sinensis* (L.) Kuntze

Shill Et Al. [193] demonstrated that *Camellia sinensis* leaf extract (CSLE) exhibits distinct microbiome-modulating properties through selective immunological activity. In Vitro studies showed that CSLE enhances the activity of signaling molecules released by dendritic cells, thereby improving the elimination of pathogenic microorganisms while preserving commensal microbes typical of the skin. This targeted effect supports the microbial balance of the epidermis, reducing the risk of dysbiosis and reinforcing natural defense mechanisms. The authors emphasize that this is the first report documenting the immunomodulatory influence of CSLE on the skin microbiome, referred to as “dermal-microbiome immunology” [193].

##### *Punica granatum* L.

Findings suggest that pomegranate may promote selective modulation of the skin microbiota by supporting the presence of beneficial species. A clinical study by Chakkalakal et al. [194] demonstrated that four weeks of supplementation with a standardized *Punica granatum* fruit extract containing 75 mg of punicalagin in healthy individuals increased the abundance of the commensal bacteria *S. epidermidis* and members of the genus *Bacillus* in the skin microbiome. These changes were accompanied by improvements in epidermal barrier function, a decrease in TEWL, a tendency toward reduced sebum secretion, and wrinkle reduction. The authors attributed these effects to the antioxidant and anti-inflammatory properties of pomegranate constituents, which help maintain a favorable microbiota while limiting pathogens [194]. Similar observations were reported by Henning et al. [195], who evaluated supplementation with *P. granatum* fruit extract (PomX) and pomegranate juice (PomJ) in clinical trials. The intervention modulated the skin microbiome by inducing shifts at the family and genus levels, though not at the phylum level. Increases in the abundance of commensal bacteria such as *S. epidermidis* and *Bacillus* spp. were most frequently observed, supporting the epidermal barrier and modulating immune responses. In the PomX group, additional changes were noted in the families Methylobacteriaceae, Aerococcaceae, and Campylobacteraceae [195].

##### *Selenicereus undatus* (Haw.) D.R.Hunt (Pitahja)

The extract of *Selenicereus undatus* (dragon fruit) may represent a valuable ingredient in cosmetics designed to support skin health and appearance by maintaining or restoring microbiota balance [196]. The natural fruit extract of *S. undatus* demonstrates skin microbiome–modulating properties. In vitro, the extract stimulated the growth of beneficial commensal species (*S. hominis*, *S. epidermidis*) while significantly reducing the abundance of pathogenic microorganisms (*S. aureus*, *C. acnes*).

In a double-blind, placebo-controlled clinical trial, 28 days of topical application of a preparation containing 1% extract increased skin microbiota diversity—particularly in older individuals—and reduced the presence of *Corynebacterium tuberculostearicum*, a species associated with unpleasant odor, inflammatory processes, and skin diseases. These microbial changes coincided with improvements in skin condition, including reduced inflammation, strengthened epidermal barrier function, increased resistance to irritants, enhanced skin tone uniformity, brightening, and wrinkle reduction [196].

##### *Solanum lycopersicum* L.

Findings by Rajkowska K. et al. [197] indicate that the addition of tomato pomace oil in a cream may support skin microbiome homeostasis and limit colonization by environmental microorganisms, potentially protecting against the adverse effects of air pollution. The study evaluated the impact of the formulation on the facial skin microbiome, analyzing changes in bacterial diversity (Shannon index) and taxonomic composition after 7 days of use compared with a base cream. It was found that the cream containing tomato oil increased the abundance of dominant commensal genera (*Staphylococcus*, *Anaerococcus*, *Cutibacterium*), particularly the beneficial *S. epidermidis*, while simultaneously reducing the prevalence of potential opportunistic pathogens (*Kocuria* spp., *Micrococcus* spp., *Veillonella* spp., *Rothia* spp.). Importantly, no elimination of key commensal species such as *C. acnes* or *Corynebacterium* spp. was observed, confirming that this ingredient helps preserve the microbiological balance of the skin [197].

##### *Myristica fragrans* Houtt.

In vitro studies conducted by Oo et al. [198] demonstrated that seed extracts of *Myristica fragrans* both the crude extract (CE) and the essential oil (EO) in combination with the commensal *S. epidermidis*, stimulate the growth of this beneficial species and induce the production of secondary metabolites with strong antimicrobial activity. In cultures, the generation of short-chain fatty acids (SCFAs) and AMPs was observed, effectively inhibiting the survival and biofilm formation of *S. aureus*. These results highlight the potential of such a combination in modulating the skin microbiome and preventing infections by promoting the dominance of commensal microorganisms [198].

##### *Ribes nigrum* L.

A polyphenolic extract from *Ribes nigrum* fruit, obtained through an enzymatic method, exhibits remarkable selectivity toward *Staphylococcus* species. In vitro *and* ex vivo studies demonstrated that at low concentrations it stimulates the growth of beneficial coagulase-negative staphylococci (CoNS), such as *S. epidermidis* (approx. 300-fold increase in CFU), while simultaneously suppressing the proliferation of pathogenic *S. aureus* strains isolated from patients with AD. In a human stratum corneum model, it was further shown that even small doses of the extract could completely reverse the unfavorable CoNS/*S. aureus* ratio, restoring commensal dominance. This effect was sustained over time, and incorporation of the extract into gel formulations additionally improved skin hydration [199,200]. In subsequent studies, Petrov Ivanković et al. [199] reported that polyphenols obtained via enzymatic extraction exhibit a higher prebiotic activity index compared to conventional extracts. They selectively promoted the growth of *S. epidermidis*, moderately inhibited *C. acnes*, and in hydrogel form improved skin hydration, reduced irritation, and enhanced dermatological compatibility [199].

#### 5.4.2. Thermal Water

Avène Thermal Spring Water (ATSW), sourced from the Montagne Noire massif in France, has been known for its therapeutic properties since the mid-18th century [201]. The unique biogeochemical and physicochemical conditions of the deep aquifer system have enabled the development of specific microbial communities. From ATSW, a novel bacterial species *Aquaphilus dolomiae* was isolated and identified [202]. A postbiotic derived from a biotechnological extract of this microorganism has demonstrated immunomodulatory, anti-inflammatory, and anti-pruritic properties, confirmed in pharmacological models of AD and chronic pruritus [203,204,205,206]. Similar properties are attributed to Vichy Volcanic Mineralizing Water, characterized by its high mineral content, which strengthens the skin barrier. It also provides a cultivation environment for *Vitreoscilla filiformis*, a bacterium considered probiotic. Extracts of this bacterium, incorporated into dermocosmetic formulations, have been shown to support the regeneration of environmentally stressed skin, accelerate epidermal renewal, and promote the maintenance of skin microbiome balance [207].

#### 5.4.3. Biosurfactants

Biosurfactants (microbially derived soaps) are another class. For example, rhamnolipid surfactants from *Pseudomonas* lower surface tension like conventional detergents but are gentler on commensals. In vitro, rhamnolipids inhibit *S. aureus*, *P. acnes* and certain fungi, yet they are well-tolerated by human keratinocytes. A blend of glycolipid surfactants selectively reduced pathogens on acne lesions without impairing overall microbiome diversity [208].

### 5.5. Animal-Derived Substances

Animal-derived substances (e.g., honey, propolis, royal jelly) have long been used for their broad antimicrobial and anti-inflammatory properties. Honey’s high osmolarity and hydrogen peroxide content inhibit many bacteria, including *S. aureus* and *P. aeruginosa*, while some commensals can tolerate or even help metabolize honey sugars.

#### 5.5.1. Honey

Honey, owing to its multifactorial antimicrobial properties, exerts a significant influence on the skin microbiome, impacting both pathogenic bacteria and the balance of commensal flora. With regard to commensal bacteria such as *S. epidermidis* and certain *C. acnes* strains, it is particularly important that honey acts selectively it suppresses the growth of opportunistic and pathogenic microorganisms which, under dysbiotic conditions, may displace beneficial microbiota. In this way, honey promotes the restoration of microbial homeostasis in the skin, which is of special relevance in diseases such as AD, where excessive colonization by *S. aureus* exacerbates inflammation and impairs epidermal barrier function [209,210,211]. Manuka honey, one of the most extensively studied types of honey, exhibits strong in vitro activity against numerous skin pathogens, including MRSA, *S. aureus*, *P. aeruginosa*, *Escherichia coli*, and *Streptococcus pyogenes*. Furthermore, it inhibits the growth of dermatophytes such as *Trichophyton rubrum*, *Microsporum canis*, and *Epidermophyton floccosum*, as well as yeasts of the genus *Candida*—though *C. albicans* displays relatively higher resistance to honey’s effects [212,213]. Thus, honey can reduce pathogens that disrupt microbiome balance without completely eradicating the skin’s resident microorganisms. Beyond its direct growth-inhibitory activity, honey also downregulates pathogen virulence factors, including *S. aureus* toxins, reduces biofilm formation, and interferes with cell–cell communication (quorum sensing), thereby diminishing pathogen virulence [214,215]. Importantly, in vitro studies have not shown the development of honey-resistant strains, which distinguishes it from antibiotics and underscores its value [209,211].

#### 5.5.2. Propolis

Propolis, a resinous substance collected by bees from tree buds and plant exudates, contains numerous bioactive compounds, primarily flavonoids (galangin, chrysin, pinocembrin), phenolic acids, and esters that account for its broad antimicrobial activity [216]. Studies have shown that propolis effectively inhibits the growth of skin pathogens, including *S. aureus* and *C. albicans*, while simultaneously supporting the formation of beneficial biofilms produced by *S. epidermidis*. These biofilms serve as a natural barrier, protecting the skin against colonization by pathogenic microorganisms [217]. Propolis also demonstrates synergism with antibiotics, enhancing their activity against pathogenic bacteria and allowing the use of lower drug doses. This, in turn, reduces the risk of microbiome imbalance and highlights propolis as a potential adjunct in therapy [218]. Moreover, reports suggest that the chemical composition of propolis varies depending on its botanical and geographical origin, which may modulate the spectrum of its activity. Moreover, reports suggest that the chemical composition of propolis, which depends on its botanical and geographical origin, may modulate the spectrum of its activity. However, in the context of the skin microbiome, its most crucial feature is the selective support of commensals while simultaneously inhibiting pathogens [219].

#### 5.5.3. Royal Jelly

Royal jelly contains 10-hydroxy-2-decenoic acid (10-HDA), major royal jelly proteins (MRJPs), and antimicrobial peptides. These compounds inhibit the growth of *C. albicans* and *S. aureus*, while their activity against commensal microorganisms is weaker, suggesting a potential role in supporting skin microbiome balance [220]. In addition, royal jelly demonstrates immunomodulatory and anti-inflammatory properties, promoting epidermal regenerative processes in both in vivo and in vitro studies [221].

#### 5.5.4. Beeswax

Beeswax, although not exhibiting strong antimicrobial activity (with only moderate effects reported against certain bacteria and fungi), primarily serves as a physical protective barrier. It reduces TEWL and stabilizes the skin microenvironment, thereby indirectly supporting the maintenance of a healthy microbiota [222].

#### 5.5.5. Pollen and Bee Bread

Bee pollen and bee bread, owing to their rich content of polysaccharides, oligosaccharides, amino acids, polyphenols (flavonoids and phenolic acids), and trace elements, may act as prebiotics in the context of the skin microbiome. These compounds represent a potential energy source for beneficial commensal bacteria such as *S. epidermidis* and non-pathogenic *C. acnes* strains, thereby supporting protection against colonization of the skin by pathogens including *S. aureus* and *C. albicans* [223]. In vitro studies have shown that bee pollen extracts promote the growth of probiotic strains (*Lactobacillus*, *Bifidobacterium*), while its polyphenols modulate the microbiota by inhibiting the proliferation of pathogenic bacteria and supporting the biofilm formation of commensal microorganisms [224,225]. Although most data concern the gut microbiome, similar mechanisms may also occur in the skin, where polysaccharides and phenolic acids can support microbial balance and reinforce the epidermal barrier through their anti-inflammatory and antioxidant effects.

#### 5.5.6. Snail Mucin

Current reports [226] suggest that snail mucins contain natural AMPs capable of selectively inhibiting pathogenic microorganisms. The mucus of *Achatina fulica* has demonstrated activity against Gram-positive bacteria (*Bacillus subtilis*, *S. aureus*) as well as Gram-negative species (*Escherichia coli*, *P. aeruginosa*). It has been hypothesized that this activity may help maintain microbiome balance by limiting pathogens while sparing commensal bacteria. However, no direct studies to date have confirmed that mucus from different snail species actually increases the abundance or activity of protective microorganisms such as *S. epidermidis* or *C. acnes*. This area remains promising but is still insufficiently validated scientifically.

#### 5.5.7. Chitosan

Chitosan is a natural polysaccharide obtained primarily from demineralized crustacean shells, exhibiting a broad spectrum of antimicrobial activity against Gram-positive and Gram-negative bacteria as well as fungi [227,228]. Its activity is associated with the cationic nature of the molecule, which enables disruption of cell membrane integrity, chelation of metal ions, and blockage of nutrient exchange, ultimately leading to bacterial cell death [227]. When applied to the skin, chitosan forms a thin protective film that retains moisture and limits pathogen penetration, thereby supporting epidermal barrier function [229]. In vitro studies have shown strong activity against *C. acnes* and *S. aureus*, with the effect being dependent on both concentration and molecular weight of the polymer [230]. Clinical studies using chitosan-impregnated fabrics in patients with AD demonstrated an increase in beneficial *Staphylococcus* populations along with a reduction in colonization by pathogenic strains, indicating its potential in modulating the skin microbiome [231]. In addition, chitosan exhibits anti-inflammatory properties, reduces the production of inflammatory mediators, and accelerates wound healing, making it an attractive candidate for use in dermocosmetics and formulations supporting the treatment of inflammatory skin diseases [229].

Table 3 clearly highlights a distinct trend: natural-origin ingredients can modulate the microbiome in a more selective manner compared to antibiotics. Many of them inhibit pathogens while sparing or in some cases even supporting—beneficial microorganisms. Plant-derived examples (such as witch hazel, green tea, and blackcurrant) and animal-derived ones (such as honey, propolis, and chitosan) demonstrate that nature provides substances with both antibiotic-like and prebiotic properties. Their application in cosmetology enables the development of products that restore skin eubiosis, for example, after disturbances or during disease, while simultaneously promoting skin care.

Each of these ingredients can be viewed through a mechanistic lens, summarized below in class-specific “mechanistic boxes”.

##### Mechanistic Boxes (By Compound Class)

β-Glucans (e.g., from oats—*Avena sativa* L.): Fermented by skin commensals (e.g., *S. epidermidis*) into lactic acid and SCFAs → low pH environment → inhibits pathogens like *S. aureus*. Also modulates immunity by promoting regulatory cytokines [156,157,158].

Oligosaccharides (e.g., inulin, fructooligosaccharides, GOS): Provide selective nutrients to commensals. Their fermentation produces organic acids (SCFAs, lactate) → acidifies microenvironment and impairs biofilm formation by pathogens. These metabolites also stimulate epidermal repair and anti-inflammatory signaling [136,140,200].

Polyphenols (e.g., catechins, flavonoids): Antioxidant and anti-inflammatory; directly disrupt microbial cell membranes or inhibit bacterial enzymes. They preferentially inhibit pathogens and often have prebiotic-like effects on commensals. Polyphenols can reinforce host defenses (AMP upregulation, cytokine modulation) [193,199,200,224].

Terpenoids/Essential Oils (e.g., thymol, eucalyptol): Lipophilic compounds that insert into microbial membranes, causing permeability and death. Broad-spectrum antimicrobial effects at certain doses. Many also modulate keratinocyte inflammation and promote barrier lipids [174].

Biosurfactants (e.g., rhamnolipids, sophorolipids): Amphiphilic molecules that solubilize microbial membranes of pathogens. Provide cleansing action while preserving commensals at proper formulation. They can also disperse biofilms [208].

Antimicrobial Peptides (AMPs, bacteriocins): Small peptides (often cationic) produced by bacteria or included in extracts. They bind to bacterial membranes and pores, causing lysis. They may also signal via host receptors (TLRs) to modulate immune responses [22,26].

Skin Lipids (ceramides, FFAs): Structural lipids (ceramides, cholesterol) support barrier integrity, indirectly stabilizing microbiota. Free fatty acids (e.g., sapienic acid from sebum) have direct antimicrobial activity: sapienic acid can inhibit *S. aureus* [19]. Lipid metabolites from microbes (e.g., short-chain fatty acids) also signal host cells to produce AMPs and tighten junctions.

Probiotics (live microbes): Compete with pathogens for nutrients and niches; produce organic acids (e.g., lactic acid) and antimicrobial peptides (bacteriocins); strengthen barrier by inducing host lipid and AMPs (ceramides, defensins); and modulate immunity (↑ IL-10, Treg induction, ↓Th17) [93,127].

Postbiotics (microbial metabolites/lysates): Contain immunomodulatory molecules (teichoic acids, polysaccharides), organic acids, and peptides. They exert anti-inflammatory effects on keratinocytes, stimulate tight junctions, and reduce pathogen virulence. For example, microbial SCFAs and peptides signal via G-protein receptors to downregulate NF-κB inflammation [55,93,165,166].

## 6. Interplay with the Gut–Skin Axis

Emerging evidence underscores the bidirectional gut–skin axis: gut microbiota influence systemic immunity and metabolite levels that affect skin homeostasis [12,265]. Oral intake of pre- or probiotics can thus have dermatological effects. For example, oral Bifidobacterium adolescentis raised systemic propionate/butyrate and ameliorated atopic dermatitis symptoms in mice [265]. Likewise, dietary fibers (inulin-type FOS) increase gut SCFAs, which circulate and promote keratinocyte differentiation and Treg function [12,265]. Some clinical trials have shown that dietary probiotics improve AD and psoriasis outcomes, presumably via immune modulation. Conversely, systemic inflammation from gut dysbiosis (e.g., IBD) often co-occurs with skin inflammation (acne, psoriasis) [12]. While a full treatment of the gut–skin axis is beyond this review, these connections justify considering systemic factors when evaluating skin microbiome interventions.

## 7. Examples of Commercial Ingredients Supporting the Skin Microbiome

Although most of this work focuses on the mechanisms and effects of natural substances, it is also worth highlighting examples of ready-to-use, commercially available active ingredients with documented skin microbiome-supporting effects. The following table (Table 4) provides a brief supplement, presenting selected preparations used in modern cosmetic formulations.

Ecoskin^®^ (INCI: Alpha-glucan oligosaccharide, *Polymnia sonchifolia* root juice, Maltodextrin, *Lactobacillus*) (Solabia) is a prebiotic–postbiotic complex obtained through a fermentation process. It is an active ingredient composed of α-glucooligosaccharides (GOS) produced by enzymatic synthesis from plant-derived raw materials, combined with a plant juice rich in β-fructooligosaccharides (FOS) extracted from yacon (*Polymnia sonchifolia*) tubers, and further enriched with probiotic bacteria of the genus *Lactobacillus* (*L. casei*, *L. acidophilus*) [266].

Oligolin^®^ (INCI: Hydrolyzed linseed extract) is a hydrolysate derived from flaxseed, concentrated in oligosaccharides. In addition to its moisturizing effect on the skin, it has been shown to exert a prebiotic activity toward beneficial skin microbiota [267].

Bioecolia^®^ (INCI: α-glucooligosaccharide) is a prebiotic with documented protective effects on the skin microbiome. It acts as a selective substrate for commensal bacteria, limiting pathogen colonization and counteracting dysbiosis. It strengthens the skin’s defenses by stimulating the synthesis of AMPs and modulating inflammatory mediators. At the same time, it protects the epidermal barrier against dehydration and microbial penetration [268].

Serenibiome^®^ (INCI: Propanediol, Glycolipids) (Solabia) is a biomimetic ingredient obtained through the biofermentation of *Pseudozyma flocculosa*. It selectively reduces the colonization of *S. aureus* without disturbing the balance of commensal bacteria. As a result, it alleviates symptoms of atopy, reduces inflammation and itching, and supports the regeneration of the epidermal barrier, making it a valuable component of cosmetics for sensitive and atopic skin [269].

Relipidium^®^ (INCI: Hydrolyzed Yeast Protein, Butylene Glycol, Pentylene Glycol) (BASF) is a fermented yeast protein hydrolysate obtained with the participation of *Lactobacillus*. It stimulates epidermal lipid biosynthesis, strengthens the skin barrier, and supports microbiome balance [270].

Hydrasensyl^®^ (INCI: Water, Pentylene Glycol, Beta-Glucan, Caprylyl Glycol) (BASF) is a β-glucan–based ingredient with prebiotic activity that helps strengthen the skin microbiome. It provides intense hydration, reduces erythema, and restores comfort to dry and sensitive skin, making it a component of barrier-repair and soothing cosmetic formulations [271].

Phytofirm^®^ Biotic (INCI: *Lactobacillus*/Soybean Ferment Extract, Pentylene Glycol, Caprylyl Glycol) (BASF) is a soybean extract fermented with *Lactobacillus plantarum*, providing probiotic activity for the skin microbiome. Rich in peptides and lactic acid, it supports skin regeneration, elasticity, and balance, making it an ingredient in anti-aging and barrier-strengthening cosmetics [272].

Lactobiotyl^®^ (INCI: Maltodextrin, *Lactobacillus* Ferment) is a postbiotic ingredient derived from the fermentation of Lactobacillus arizonensis, a bacterium adapted to dry desert conditions. It strengthens the skin barrier, supports cell renewal, and helps maintain microbiome balance. Clinical tests on the face and hands demonstrated improved hydration and radiance of dry skin in both Caucasian and Asian volunteers [273].

## 8. Research Gaps and Future Directions

Despite progress, significant gaps remain. First, multi-omics and mechanistic studies are needed. Most human studies use 16S rRNA or metagenomics but few integrate metabolomics, transcriptomics, or proteomics to reveal what microbes do. Advanced approaches (e.g., shotgun metagenomics, metabolomics of skin surface) could link compositional changes to functional shifts [12]. Longitudinal and placebo-controlled trials with such endpoints will clarify causal pathways.

Second, strain and context specificity must be addressed. Many findings show that species are too coarse: e.g., acne-associated *C. acnes* have multiple phylotypes with opposite effects. Detailed strain-level analysis is crucial for targeted therapy. Similarly, the efficacy of a probiotic or postbiotic depends on the exact strain(s) used. Future work should standardize strains, dosages, and formulations, and compare them head-to-head.

Third, long-term safety and efficacy studies are lacking. Most trials are short (weeks) and often in adults. We need data on chronic application (months to years), especially for leave-on products. Concerns include potential for sensitization, effects on skin resistome (antibiotic resistance genes), and systemic absorption. Pediatric and immunocompromised populations warrant careful evaluation. The interplay of topical treatments with concurrent therapies (antibiotics, steroids, etc.) is also understudied.

Fourth, the gut–skin axis requires deeper exploration. While conceptually recognized, few dermatology trials include gut-microbiome analysis or systemic metabolite measures. Future research should assess how oral interventions (diet, probiotics) influence skin outcomes via the gut.

Finally, improved standardization of outcomes is needed. Validated clinical endpoints (beyond surrogate biomarkers) and consideration of formulation factors (pH, vehicle, preservative compatibility) will enhance reproducibility. For example, the preservative or surfactant in a cream may negate a prebiotic effect.

In summary, future skin microbiome research should leverage multi-omics technologies to uncover functional mechanisms, test defined microbial strains and compounds in rigorously controlled trials, and monitor long-term outcomes. Advances in omics will refine our understanding of how bioactive ingredients reshape the skin ecosystem, and will guide personalized microbiome-based dermatology.

## 9. Conclusions

Cutaneous eubiosis arises from a balance of niche chemistry, host defense, and microbe-microbe interactions. In conditions like acne, AD, psoriasis, and chronic wounds, dysbiotic shifts (barrier defects, biofilms, cytokine imbalances) perpetuate disease. Our review highlights that selective modulation—rather than indiscriminate killing—is increasingly feasible. Botanicals (polyphenols, terpenes), bee products, prebiotics (β-glucans, oligosaccharides), probiotics, and postbiotics can reduce pathogen virulence, restore acidity and ceramide levels, and bolster colonization resistance. Early clinical studies already show improvements in hydration, TEWL, erythema, and lesion counts with microbiome-friendly formulations.

**Figure 1:**
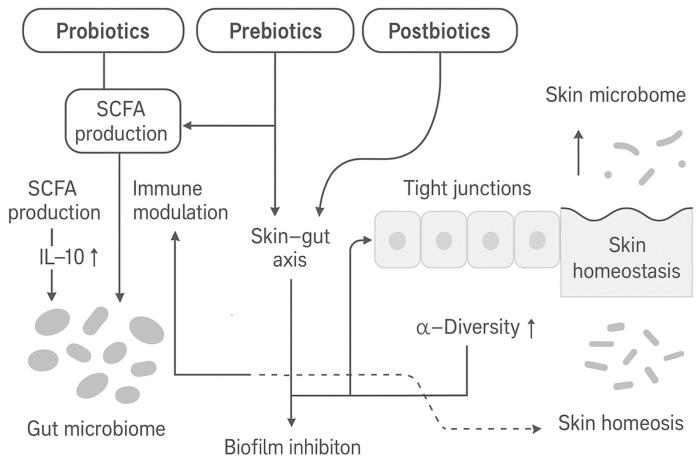
Proposed mechanisms of skin microbiome modulation by bioactive compounds. SCFA—short-chain fatty acids; IL-10—interleukin-10, ↑ increase.

The mechanistic Figure 1 illustrates how different ingredient classes influence the skin ecosystem. For example, β-glucans and oligosaccharides (prebiotics) are fermented by commensal bacteria into lactic acid and short-chain fatty acids (SCFAs), lowering skin pH and inhibiting pathogens. Polyphenol-rich extracts disrupt pathogen membranes and reduce oxidative stress; terpenoids from essential oils similarly disrupt microbial membranes. Biosurfactants (e.g., rhamnolipids) solubilize pathogen membranes during gentle cleansing. Probiotic bacteria (*Lactobacillus*, *Bifidobacterium*) compete for nutrients, secrete organic acids and bacteriocins, and stimulate host immune tolerance (↑ IL-10, Treg) [127]. Postbiotics (fermentation-derived metabolites, lysates) supply additional antimicrobial peptides and immune-activating molecules that reinforce barrier integrity. Collectively, these mechanisms preserve commensals, suppress pathogens, and modulate host inflammatory and barrier responses.

In addition, the table below (Table 5) summarizes the mechanistic pathways connecting microbiome-based interventions to biological targets, biomarkers, and clinical outcomes.

Looking ahead, translational progress hinges on standardization. Studies must specify strain-level identity, dose–response, and vehicle conditions. Compatibility with formulation components (preservatives, pH) must be tested to ensure activity. Well-designed trials with clinical endpoints, integrated with conventional therapies, will determine which microbiome-supportive interventions yield durable benefits. Ultimately, multi-omics approaches and long-term safety data will advance the field towards personalized microbiome-based dermatology.

## Figures and Tables

**Table 2:** Skin microbiome-modulating substances—classification according to ISAPP.

Category	Mechanism of Action	Examples
Probiotics	Compete with pathogens for niches and nutrients; produce bacteriocins and organic acids; strengthen the epidermal barrier (ceramides, AMPs); modulate immunity (↑ IL-10, ↓ Th17) [128].	*Lactobacillus rhamnosus*, *L. plantarum*, *L. johnsonii*, *Bifidobacterium breve*, *Streptococcus thermophilus*, *Staphylococcus hominis*, *Roseomonas mucosa* [133].
Prebiotics	Provide selective nutrients for commensals (e.g., *S. epidermidis*, non-pathogenic *C. acnes*); support diversity; maintain acidic pH and lipid balance [136].	Inulin, β-glucan, oligosaccharides, FOS, GOS, COS, HMOs, algal polysaccharides [136]
Postbiotics	Exert anti-inflammatory and immunomodulatory effects; strengthen the skin barrier; reduce pathogen colonization; improve hydration [161,162,163].	Antimicrobial peptides, lactic acid and SCFAs (*Cutibacterium*), bacterial polysaccharides (EPS) [161,162,163].

**Table 3:** Natural plant- and animal-derived substances modulating the skin’s microbiome.

	Mechanism of Action on the Microbiome	Cosmetological/Dermatological Applications
*Symphytum officinale* root	The extract maintains microbiota diversity; skin bacteria metabolize its components, producing anti-inflammatory metabolites that support the skin barier [186].	Comfrey leaves and roots are used in concentrations between 5% and 20% in creams, mainly for healing superficial wounds and it is topical use in skin conditions [232,233]. According to the European cosmetic ingredient database (CosIng) extracts of this plant serve multiple functions in cosmetics, including, anti-seborrheic; skin conditioning; soothing [234,235].
*Calendula officinalis*	It exhibits antibacterial activity; inhibits the growth of *C. acnes*. It may also reduce the excessive growth of *S. epidermidis* [187,188]. Ethanolic and methanolic extracts exhibited inhibitory effects against *C. albicans* [190].	According to the (CosIng), extracts of this plant serve multiple functions in cosmetics, including skin conditioning, emollient, skin protection, fragrance, perfuming, and humectant [236,237,238]. *Calendula* oil cream has been reported to protect the skin from UV radiation when used in sunscreen formulations and to help maintain the natural pigmentation of the skin [239].
*Arnica montana*	Suggestions of a beneficial effect on the composition of the microbiome [186].	According cosing: *Arnica montana* extract used in cosmetics mainly as a skin-conditioning and soothing agent [240]. *Arnica montana* flower extract functions as a fragrance and perfuming ingredient, sometimes also contributing to skin protection [241]. *Lactobacillus/Arnica montana* Flower Ferment Filtrate—obtained by fermenting *Arnica montana* flowers with *Lactobacillus*; acts mainly as a humectant and skin-conditioning agent [242].
*Centella asiatica*	The extract shows broad antibacterial activity, particularly against *S. aureus*. It has antioxidant and anti-inflammatory properties and strengthens the skin barrier, helping to maintain microbiological homeostasis [191].	According cosing: *Centella asiatica* extract used in cosmetics for cleansing, skin conditioning, smoothing, soothing, and tonic functions [243]. Hydrolyzed *C. asiatica* extract provides antioxidant and skin-conditioning (humectant) effects, supporting hydration and protection [244]. *C. asiatica* Leaf Extract—functions mainly as a skin-conditioning agent [245]. *Lactobacillus*/*Centella asiatica* extract ferment extract—obtained by fermenting *Centella asiatica* with *Lactobacillus*; acts as a miscellaneous skin-conditioning ingredient [246].
*Hamamelis virginiana*	Rich in tannins, the extract acts selectively: it inhibits the growth of pathogenic bacteria, reduces biofilm formation and toxin production (particularly against pathogenic strains of *S. aureus*, *S. epidermidis*, and *Enterococcus*). At the same time, it supports the growth of beneficial probiotic bacteria such as *Lactobacillus plantarum*, protecting them against oxidative stress [192].	According cosing: *Hamamelis virginiana* bark/twig extract—used in cosmetics mainly as an astringent and skin-conditioning ingredient [247]. *H. virginiana* water—functions as an astringent, hair-conditioning, skin-conditioning, and soothing agent [248].
*Camellia sinensis*	*Camellia sinensis* leaf extract rich in immunomodulating polyphenols; stimulates skin dendritic cells to produce signals that enhance pathogen clearance while preserving commensal microbes [193].	According Cosing: *Camellia sinensis* Leaf Extract—multifunctional cosmetic ingredient with antimicrobial, antioxidant, and astringent effects. Acts as a skin-conditioning agent (emollient, humectant), provides a fragrance, and is used in oral care. It also supports skin barrier function, protection, and has potential as a tonic and UV absorber [249]. *Lactobacillus/Camellia sinensis* Extract Ferment Extract—obtained by fermenting *Camellia sinensis* extract with *Lactobacillus*; functions as a miscellaneous skin-conditioning and skin-protecting ingredient [250].
*Punica granatum*	Pomegranate extract (standardized to punicalagin), when taken orally, modulates the skin microbiome. After 4 weeks of supplementation, an increase in the abundance of commensal *S. epidermidis* and *Bacillus* was observed. The antioxidant and anti-inflammatory components of pomegranate promote beneficial flora and limit pathogenic microorganisms [194,195].	Saccharomyces/*Punica Granatum* Fruit Ferment Filtrate—filtrate obtained by fermenting *Punica granatum* fruit with *Saccharomyces*; used in cosmetics as a skin-conditioning ingredient [251]. *Punica Granatum* Extract—extract of the whole pomegranate plant; provides antioxidant activity and skin-protecting effects [252].
*Selenicereus undatus*	The fruit extract exhibits microbiome-balancing effects. In vitro, it promotes the growth of commensal bacteria (*S. epidermidis*, *S. hominis*) while inhibiting pathogens (*S. aureus*, *C. acnes*). In a clinical study, a cream containing the extract increased microbiome diversity (Faith’s diversity +20% vs. placebo) [196].	There is no information in the CosIng database regarding *Hylocereus undatus* (pitahya, dragon fruit). The extract appears beneficial in reduced redness, improved skin barrier function (−13% TEWL), enhanced radiance (+11% ITA), and evened out pigmentation [196].
*Lycopersicon esculentum* oil	An example of cosmetic upcycling, showing an increase in dominant commensals (including *Staphylococcus* spp., *Anaerococcus*, *Cutibacterium*), particularly *S. epidermidis*, while inhibiting (e.g., *Kocuria*, *Micrococcus*, *Veillonella*, *Rothia*) [197].	There is no information in the CosIng database regarding oil from tomato pomace
*Myristica fragrans* (nutmeg)	The extract stimulates the proliferation of *S. epidermidis*, inducing the production of secondary metabolites—SCFAs and AMPs—which inhibit the survival and biofilm formation of *S. aureus* [198].	According Cosing: *Myristica fragrans* seed extract functions in cosmetics primarily as a skin-conditioning ingredient, acting both as an emollient and a humectant, helping to soften the skin and improve its hydration [253].
*Ribes nigrum*	The enzymatically obtained polyphenol extract stimulates the growth of beneficial coagulase-negative staphylococci (*S. epidermidis*) while inhibiting pathogenic *S. aureus*. In an ex vivo stratum corneum model, even low doses of the extract were able to fully restore the favorable balance, re-establishing the dominance of commensal *Staphylococcus* [199,200].	According to CosIng, *Ribes nigrum* leaf extract functions as a skin-conditioning ingredient [254], while *R. nigrum* fruit extract is listed with multiple functions, including astringent, skin-conditioning (emollient and general), and perfuming [255].
Mel/Honey	Selectively inhibits *S. aureus*, *Streptococcus*, Gram-negative rods, dermatophytes and yeasts, while sparing *S. epidermidis* and some *C. acnes* strains. In AD, reduces excessive *S. aureus* colonization and inflammation; lowers pathogen virulence by decreasing toxin production and biofilm formation [209,210,211].	According to CosIng, Honey extract functions as a skin-conditioning ingredient, (emollient and humectant and moisturizing), and flavoring [256]
Propolis	It has antiseptic properties: inhibits the growth of *S. aureus* and *C. albicans*, while supporting beneficial biofilms formed by *S. epidermidis*. Propolis also acts synergistically with antibiotics [216,217,219].	According to CosIng, Propolis Extract functions as a skin-conditioning ingredient [257]. The modified forms show additional properties: Saccharomyces/Propolis Ferment Extract listed as antimicrobial, antioxidant, humectant, and skin-conditioning [258]. Lactobacillus/Propolis Ferment Extract classified under miscellaneous skin-conditioning functions [259].
Royal jelly	It inhibits the growth of pathogenic microorganisms (*C. albicans*, *S. aureus*), while showing weaker effects on commensal flora, suggesting that it may support microbiome balance primarily by targeting pathogens [220,221].	According to CosIng, Royal Jelly Extract is classified as a skin-conditioning ingredient [260].
Beeswax/*Cera alba*	It forms a hydrophobic barrier that reduces TEWL and stabilizes the skin microenvironment, indirectly supporting the microbiome by maintaining hydration and barrier integrity, which strengthens defense against opportunistic microbes [222].	According to CosIng, Beeswax [261]/(*Cera alba*) [262] serves multiple functions in cosmetics: it acts as a binding agent, emulsion stabiliser, fragrance/perfuming ingredient, skin-conditioning agent (including emollient action), viscosity-controlling agent, surfactant-emulsifier, and film-forming component.
Bee pollen and bee bread	Provide energy for beneficial skin bacteria (*S. epidermidis*, non-pathogenic *C. acnes*), supporting protection against pathogens. In vitro, extracts promote growth of probiotic strains (*Lactobacillus*, *Bifidobacterium*) and modulate microbiota by inhibiting pathogens while supporting commensal biofilms. Analogous mechanisms may occur on the skin, where pollen components reduce inflammation and oxidative stress, strengthening the barrier and microbiome balance [223].	According to CosIng, Bee Pollen Extract is classified as a miscellaneous skin-conditioning, antimicrobial, antioxidant, and skin-conditioning (emollient) ingredient, while Bee Pollen Vesicles are listed as skin-protecting [263,264].

**Table 4:** Commercial cosmetic ingredients supporting the skin microbiome. (manufacturer information).

Trade Name	Ingredients	Mode of Action	Cosmetic Application
Ecoskin^®^ Solabia	α-GOS, β-FOS (yacon), *L. casei*, *L. acidophilus*	Substrate for commensals, pathogen reduction, barrier strengthening	Moisturizing creams, baby care, serums for dry and sensitive skin [266]
Oligolin^®^ BASF	Flaxseed hydrolysate (oligosaccharides)	Prebiotic, improves hydration, inhibits skin matrix-degrading enzymes	Anti-aging, firming serums, moisturizing creams [267]
Bioecolia^®^ Solabia	α-GOS	Selective support of commensals, AMP stimulation, pathogen inhibition	Products for sensitive and dry skin [268]
Serenibiome^®^ Solabia	Ferment *Pseudozyma flocculosa*	Inhibits *S. aureus*, does not affect *S. epidermidis*, reduces inflammation and itching	Products for sensitive and dry skin [269]
Relipidium^®^ BASF	Yeast protein hydrolysate fermented by *Lactobacillus*	Strengthens the barrier, supports lipid biosynthesis, regenerates the skin ecosystem	Nourishing creams, barrier creams, anti-aging products [270]
Hydrasensyl^®^ BASF	β-glucan	Prebiotic	Moisturizing serums, creams for dry and sensitive skin [271]
Phytofirm^®^ Biotic BASF	Soy extract fermented by *Lactobacillus plantarum*	Rich in peptides and lactic acid, strengthens the skin microbiota	Probiotic cosmetics, anti-aging, barrier products [272]
Lactobiotyl^®^ Silab	*Lactobacillus arizonensis* ferment	Postbiotic	Moisturizing serums, creams for dry and sensitive skin [273]

**Table 5:** Mechanistic pathways linking microbiome-based interventions with biological targets, biomarkers, and clinical outcomes.

Intervention or Compound	Primary Biological Target	Mechanistic Pathway (MoA)	Biomarkers/Molecular Effects	Expected Clinical Outcome	Ref.
Short-chain fatty acids (SCFA: acetate, propionate, butyrate)	HDAC inhibition → immune regulation	SCFA → HDAC ↓ → NF-κB ↓ → ↑ Treg/↓ Th17-IL-23 axis	↓ IL-6, ↓ TNF-α, ↓ CRP; normalized inflammatory profile	↓ Inflammation; improved skin condition and PASI reduction	[274]
Postbiotic lotion with heat-inactivated *Lactobacillus johnsonii* NCC 533	Pathogen exclusion (competitive inhibition)	↓ *S. aureus* adhesion; rebalanced surface microbiota	↓ *S. aureus* load; normalized pH; ↑ barrier integrity	Improved AD symptoms; ↓ colonization recurrence	[275]
Sonicated *Streptococcus thermophilus* (cream)	Lipid metabolism and barrier function	Bacterial enzymes → ↑ ceramide synthesis in SC	↑ Ceramides; ↓ TEWL	Enhanced barrier; ↓ dryness and irritation	[217]
Oral probiotic mix (*B. longum* CECT 7347 + *B. animalis* ssp. lactis CECT 8145 + *L. rhamnosus* CECT 8361)	Gut–skin axis; systemic immune homeostasis	Modulation of gut microbiota → SCFA ↑ → HDAC ↓ → NF-κB ↓	↑ *Collinsella*, ↑ *Lactobacillus*, ↓ *Micromonospora*, ↓ *Rhodococcus*; ↓ pro-inflammatory cytokines	↑ PASI responders (66.7% vs. 41.9%); ↓ CRP, ↓ IL-6, ↓ TNF-α	[274]
Topical LAB formulations for acne	Ecological balance in pilosebaceous unit	LAB → competition with *C. acnes*; AMP induction	↓ *C. acnes* density; ↑ endogenous AMP; ↓ IL-8	↓ Number of acne lesions; improved skin texture	[275]
Microbiome-friendly formulations (non-viable)	Barrier and microbiome homeostasis	Maintenance of pH 5–5.5; ↓ biofilm formation; ↑ AMP	Stable microbiota; balanced skin pH; preserved TEWL	Long-term tolerance; ↓ flare frequency	[71]

SCFA—Short-Chain Fatty Acids; HDAC—Histone Deacetylase; NF-κB—Nuclear Factor kappa-light-chain-enhancer of activated B cells; Treg—Regulatory T cells; Th17—T helper 17 cells; CRP—C-reactive protein; AMP—Antimicrobial Peptides; TEWL—Transepidermal Water Loss; AD—Atopic Dermatitis; PASI—Psoriasis Area and Severity Index; LAB—Lactic Acid Bacteria, ↑ increase; ↓ decrease; → resulting effect.
